# The Prognostic Significance of Low-Frequency Somatic Mutations in Metastatic Cutaneous Melanoma

**DOI:** 10.3389/fonc.2018.00584

**Published:** 2019-01-04

**Authors:** Xiaobei Zhao, Paul Little, Alan P. Hoyle, Guillaume J. Pegna, Michele C. Hayward, Anastasia Ivanova, Joel S. Parker, David L. Marron, Matthew G. Soloway, Heejoon Jo, Ashley H. Salazar, Michael P. Papakonstantinou, Deeanna M. Bouchard, Stuart R. Jefferys, Katherine A. Hoadley, David W. Ollila, Jill S. Frank, Nancy E. Thomas, Paul B. Googe, Ashley J. Ezzell, Frances A. Collichio, Carrie B. Lee, H. Shelton Earp, Norman E. Sharpless, Willy Hugo, James S. Wilmott, Camelia Quek, Nicola Waddell, Peter A. Johansson, John F. Thompson, Nicholas K. Hayward, Graham J. Mann, Roger S. Lo, Douglas B. Johnson, Richard A. Scolyer, D. Neil Hayes, Stergios J. Moschos

**Affiliations:** ^1^Lineberger Comprehensive Cancer Center, The University of North Carolina at Chapel Hill, Chapel Hill, NC, United States; ^2^Department of Biostatistics, Gillings School of Public Health, The University of North Carolina at Chapel Hill, Chapel Hill, NC, United States; ^3^Division of Hematology/Oncology, Department of Medicine, The University of North Carolina at Chapel Hill, Chapel Hill, NC, United States; ^4^Division of Surgical Oncology, Department of Surgery, The University of North Carolina at Chapel Hill, Chapel Hill, NC, United States; ^5^Melanoma Program, The University of North Carolina at Chapel Hill, Chapel Hill, NC, United States; ^6^Department of Dermatology, The University of North Carolina at Chapel Hill, Chapel Hill, NC, United States; ^7^Department of Cell Biology & Physiology, Histology Research Core Facility, The University of North Carolina at Chapel Hill, Chapel Hill, NC, United States; ^8^Division of Dermatology, Department of Medicine, Jonsson Comprehensive Cancer Center, University of California, Los Angeles, Los Angeles, CA, United States; ^9^Melanoma Institute Australia, The University of Sydney, Sydney, NSW, Australia; ^10^Queensland Institute of Medical Research-QIMR Berghofer Medical Research Institute, Herston, QLD, Australia; ^11^Department of Medicine, Vanderbilt-Ingram Cancer Center, Nashville, TN, United States

**Keywords:** The Cancer Genome Atlas Project, cutaneous melanoma, next generation sequencing, RNA sequencing, prognostic significance, UV signature, RAC1, SPEN

## Abstract

**Background:** Little is known about the prognostic significance of somatically mutated genes in metastatic melanoma (MM). We have employed a combined clinical and bioinformatics approach on tumor samples from cutaneous melanoma (SKCM) as part of The Cancer Genome Atlas project (TCGA) to identify mutated genes with potential clinical relevance.

**Methods:** After limiting our DNA sequencing analysis to MM samples (*n* = 356) and to the CANCER CENSUS gene list, we filtered out mutations with low functional significance (snpEFF). We performed Cox analysis on 53 genes that were mutated in ≥3% of samples, and had ≥50% difference in incidence of mutations in deceased subjects versus alive subjects.

**Results:** Four genes were potentially prognostic [*RAC1, FGFR1, CARD11, CIITA*; false discovery rate (FDR) < 0.2]. We identified 18 additional genes (e.g., *SPEN, PDGFRB, GNAS, MAP2K1, EGFR, TSC2*) that were less likely to have prognostic value (FDR < 0.4). Most somatic mutations in these 22 genes were infrequent (< 10%), associated with high somatic mutation burden, and were evenly distributed across all exons, except for *RAC1* and *MAP2K1*. Mutations in only 9 of these 22 genes were also identified by RNA sequencing in >75% of the samples that exhibited corresponding DNA mutations. The low frequency, UV signature type and RNA expression of the 22 genes in MM samples were confirmed in a separate multi-institution validation cohort (*n* = 413). An underpowered analysis within a subset of this validation cohort with available patient follow-up (*n* = 224) showed that somatic mutations in *SPEN* and *RAC1* reached borderline prognostic significance [log-rank favorable (*p* = 0.09) and adverse (*p* = 0.07), respectively]. Somatic mutations in *SPEN*, and to a lesser extent *RAC1*, were not associated with definite gene copy number or RNA expression alterations. High (>2+) nuclear plus cytoplasmic expression intensity for SPEN was associated with longer melanoma-specific overall survival (OS) compared to lower (≤ 2+) nuclear intensity (*p* = 0.048). We conclude that expressed somatic mutations in infrequently mutated genes beyond the well-characterized ones (e.g., *BRAF, RAS, CDKN2A, PTEN, TP53*), such as *RAC1* and *SPEN*, may have prognostic significance in MM.

## Introduction

The role of genetic aberrations in SKCM development and progression is unquestionable (1). Several studies in primary melanomas have shown the prognostic significance of frequent somatic mutations, such as *NRAS* and *BRAF* (2–4). Other studies have been more heterogeneous with respect to inclusion of both primary and metastatic melanoma samples (5, 6). Other genes have more complex genetic and epigenetic aberrations; therefore, non genetic-based assays have been used to assess prognostic significance (7). Even if studies are focused in metastatic melanoma (MM) specimens and for the most abundant *BRAF* and *NRAS* mutations, the prognostic significance in MM is less understood and, in some cases, controversial (8–10). Advances in next generation sequencing methodology have inspired the development of targeted sequencing mutation panels to assist clinicians toward personalized treatment decisions (11, 12). It is now commonplace that medical oncologists order targeted sequencing panels to identify genetic aberrations that may be predictive of response to anticancer therapies. With the exception of hematologic malignancies (13), however, sequencing panels have been infrequently used in solid tumors to assess prognosis. Is it possible that other, less well-characterized mutated genes are important for the understanding of genetics in MM as well as prognosis?

In this study, we explored the prognostic significance of somatic mutations in the TCGA SKCM cohort. Given the high somatic mutation burden in SKCM (8), we focused on mutations with intermediate/high functional impact. We have restricted our analysis to samples procured from MM for three reasons: (1) metastatic tumors are more likely to have mutations in driver genes (14); (2) passenger mutations that are associated with ultraviolet signature may be significantly less in MM (15); and (3) although not all patients with primary melanoma will succumb to their disease, patients with MM have a worse prognosis. Finally, we explored the clinical significance of our findings on genetic aberrations of MM in an independent cohort. We have identified that genetic aberrations in less characterized genes, such as *RAC1* and *SPEN*, may have prognostic significance.

## Patients and methods

### The TCGA Cohort

#### Patients, Clinical Data, and Molecular Classification

Clinical, pathologic, and follow-up data were retrieved from the TCGA (Supplemental Material). Follow-up time from both the original diagnosis of melanoma (OS-original diagnosis) and from time-to-specimen collection to latest follow-up (OS-specimen collection) were recorded. Each specimen was subsequently classified according to the previously reported molecular and gene expression profiling classifications (Supplemental Material) (8).

#### Next Generation Sequencing

The Variant Call Format (VCF) files were retrieved from the TCGA access-controlled data portal (https://cbiit.cancer.gov) using the reference GRCh37/hg19 genome assembly. Somatic mutation calls were annotated by the UNCseq™ pipeline (version 2016.07): snpEFF (v4.3) (16) was used to annotate the variant calls to ascertain gene information [gene id, HGVS_C for Variant in Human Genome Variation Society (HGVS) DNA notation; HGVS_P for Variant in HGVS protein notation] and functional impact [IMPACT for HIGH, MODERATE, LOW, MODIFIER, EFFECT for Effect in Sequence Ontology terms, http://snpeff.sourceforge.net/SnpSift.html]. The variants were also annotated with the Catalog of Somatic Mutations in Cancer (COSMIC, v77) and ExAC (v0.3) to obtain the allele frequency of the variants.

#### Identification of Mutations With Potentially High Biological Impact

Multi-step filters for somatic mutation calling were applied to identify non-synonymous mutations in potentially important genes (Supplemental Material). To ensure a high quality mutation calling, only mutations with mutant allele frequency (MAF) ≥5% and mutated allele count (MAC) ≥5 were kept (Figure S1). To further refine the gene list, the following clinically driven decisions were made: first, multiple mutations per gene per given specimen were counted once; second, only genes mutated in ≥3% of the total number of patient samples were considered [*n* = 128 (OS-original diagnosis analysis), *n* = 133 (OS-specimen collection analysis)]; third, only genes that were more or less frequently mutated in deceased vs. living subjects at the time of last follow-up were considered. By convention, these genes were defined by the fold of the mean frequency of samples mutated in deceased vs. living patients ($\text{x}=\frac{\frac{\text{No pts with a given mutated gene in deceased pts}}{\text{ Deceased pts}}\;}{\frac{\text{No pts with a given mutated gene in living pts}}{\text{Living pts}}}$) which should be either ≥1.5 or ≤ 0.67. Time to last follow-up was not taken into consideration at this stage of gene list refinement.

Univariate Cox regression analysis was subsequently performed on the final list of mutated genes to investigate the impact of the mutation frequency for each gene on OS. The null hypothesis was that the mutation frequency for each gene does not correlate with OS. Mutated genes with per gene-adjusted FDR ≤ 0.4 were considered promising.

Both DNA and RNA sequencing reads [per sample binary version of SAM files (BAM) against the hg19 assembly] were obtained to perform UNCeqR (v0.05) analysis (17), and confirm the somatic mutations of prognostically important genes.

### The Multi-Institution Validation Cohort for the TCGA Data

This multi-institution cohort consists of published datasets whose MM tumors were subjected to whole exome (18–20), genome (21), or targeted panel sequencing (22). Updated follow-up data were obtained from each dataset, if possible (18, 19, 21). We also added the MM cohort from the University of North Carolina at Chapel Hill (UNC-CH), whose tumors underwent targeted panel sequencing (IRB protocol 11-1115) (23), and clinical data were collected (IRB protocol 16-2959; the UNC-CH UNCseq™ MM cohort). Only non-synonymous somatic mutations identified in tumor samples procured from MM of cutaneous or unknown primary (i.e., no cell lines established from tumors) were included.

### The UNC-CH Metastatic Melanoma Cohort to Investigate SPEN Protein Expression in Metastatic Melanoma

To investigate protein expression of SPEN in relation to several clinical and histopathologic features in MM, we performed single-color immunohistochemical analysis of the SPEN protein expression in MM tissue samples from patients who were treated at the UNC-CH Melanoma Program. Collection of MM tumor tissues, clinicopathologic data, and clinical follow-up were allowed under the UNC-CH IRB approved protocol 09-1737. These MM tissue samples were different from those included in the UNC-CH UNCseq™ MM cohort, which was described above. Representative 0.7-mm diameter tissue cores from each MM specimen were spotted in duplicates or triplicates in the UNC-CH MM tissue microarray (TMA).

A commercially available anti-SPEN antibody (polyclonal rabbit, product #HPA-15825, Lot# A105440, Sigma-Aldrich, St. Louis, MO) (24) was optimized in normal human tissues and a nevus-primary melanoma TMA prior to staining the UNC-CH MM TMA. Briefly, 5 μm-thick tissue sections from the UNC-CH MM TMA were baked at 60°C for 90 min followed by heat-induced epitope retrieval using HIER Buffer L (Thermo Scientific, TA-135-HBL, Waltham, MA). Tissues were blocked using 10% normal goat serum for 1 h at room temperature, then incubated with the anti-SPEN antibody overnight at 4°C. Following incubation with a biotinylated goat anti-rabbit IgG (1:500; Jackson ImmunoResearch Laboratories Inc., 111-065-144, West Grove, PA) for 60 min at room temperature, tissues were treated with ABC-AP (Vector Laboratories, AK-5000, Burlingame, CA) and Impact Vector Red (Vector Laboratories, SK-5105). Finally, the tissues were counterstained with hematoxylin (Thermo Scientific, 6765003), dehydrated, cleared, and coverslipped using DPX (Electron Microscopy Sciences, 13512, Hatfield, PA).

Histopathologic analysis was performed using the 0, 1+ (< 25% of melanoma cells with nuclear stain), 2+ (25–80% of melanoma cells with nuclear stain), and 3+ (>80% of melanoma cells with strong stain) semiquantitative scale (PBG). Each tissue core was also evaluated for the presence or absence of any small-diameter mononuclear cells with stippled chromatin and indistinctive cytoplasm suggestive of lymphocytes. Contingency tables and the chi-square test were used to assess the association between expression intensity of SPEN protein by melanoma cells and the presence vs. absence of tumor-infiltrating lymphocytes (R project, www.r-project.org). Kaplan-Meier curves were constructed using GraphPad Prism (v 8.0, GraphPad, La Jolla, CA) to estimate the melanoma-specific overall survival (OS) in patients with high (>2+) nuclear plus cytoplasmic nuclear-only signal vs. high (>2+) nuclear-only signal vs. low (≤ 2+) expression of SPEN protein by melanoma cells. Given the multiple replicates per patient, the average of the expression intensity was considered. In addition, a specimen was classified as having both nuclear and cytoplasmic signal if at least one of the replicate cores exhibited nuclear plus cytoplasmic localization for the SPEN protein.

## Results

### Bioinformatics Analysis of Somatic Mutations in TCGA MM Samples

**Figure 1:**
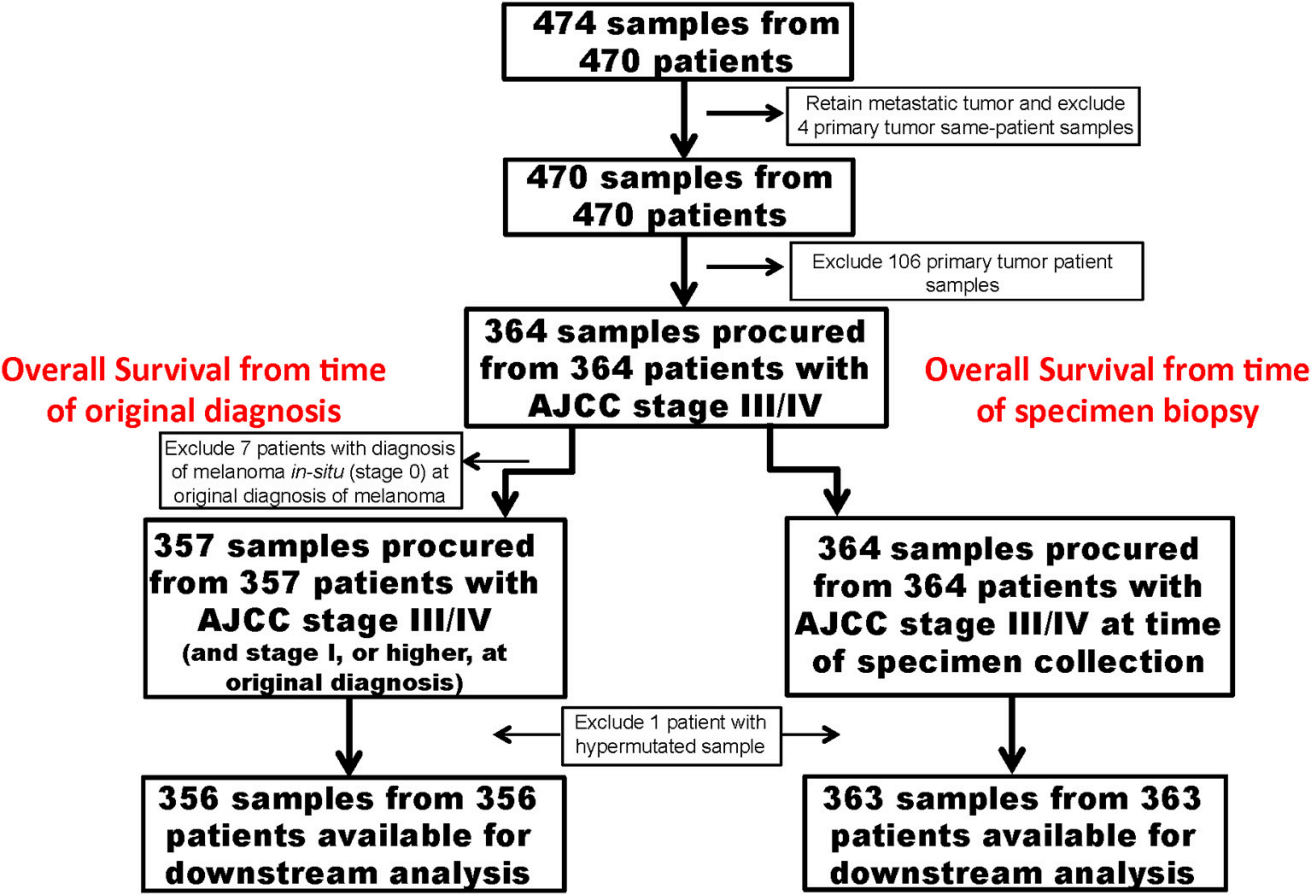
Strategy to identify important somatic mutations in the TCGA SKCM cases. Flow diagram shows important clinical steps, such as removal of cases procured from patients with primary melanoma and lack of long-term follow-up. For the OS-original diagnosis analysis, we excluded patients who were originally diagnosed with AJCC stage 0. For the OS-specimen collection analysis, time was calculated from time of specimen collection.

**Figure 2:**
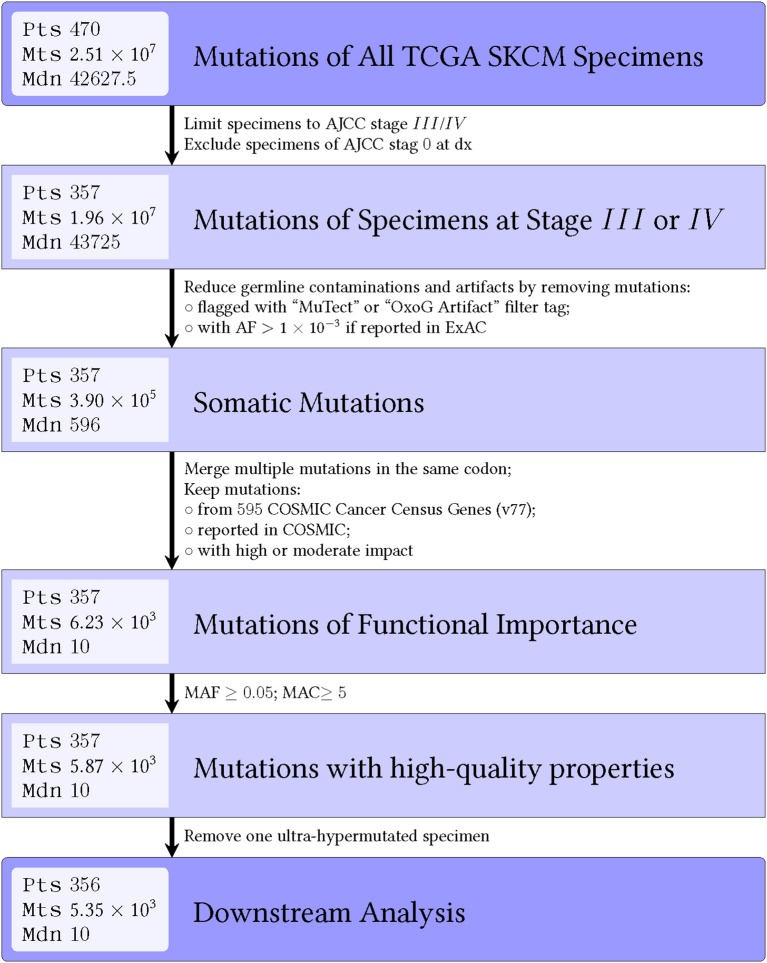
Flow diagram shows number of mutations that are filtered out during each step for the OS-original diagnosis analysis. Similar results were seen for the OS-specimen collection analysis. Abbreviations: pts, patients; mts, mutations; mdn, median; AJCC, American Joint Committee on Cancer; dx, diagnosis; AF, allele frequency; OxoG, oxidative artifact from oxidation of guanine to 8-oxoguanine during DNA library preparation; COSMIC, catalog of somatic mutations in cancer.

Figures 1, 2 show details of our filtering strategy. 25,102,889 mutations, both somatic and presumed germline, were identified in 474 tumors. For the OS-original diagnosis survival analysis, we excluded samples from stage 0 melanoma, whereas for the OS-specimen collection analysis, we excluded patients with a discrepant biopsy date. For the OS-original diagnosis analysis, 5,351 mutations (0.22% total; 5,285 single nucleotide variations, 37 dinucleotide variants, and 29 insertions/deletions) in 537 genes were found in 356 tumors [median, 10 mutations, ~95% confidence interval (~95CI) 9–11 mutations; range, 0–132 mutations]. At a median follow-up of 53.0 months (~95CI, 47.2–59.1 months), 185 (51.8%) patients were deceased. We observed similar findings in the OS-specimen collection analysis [5,481 mutations in 541 genes were found in 363 tumor specimens; median follow-up 53.0 months (~95CI, 47.8–59.2 months); 190 (52.3%) patients were deceased]. Table 1 shows demographics, molecular, and gene expression classification of the 356 MM patients.

**Table 1:** Patient characteristics of the TCGA SKCM MM tissue cohort (*n* = 356).

**Characteristics**	**Total (*n* = 356)**
**AJCC (at specimen procurement)**	
III	291
IV	65
**AJCC (at original diagnosis)**	
I	75
II	75
I or II not-otherwise specified	13
III	139
IV	20
Not Available	34
**sex**	
Male	222
Female	134
**Race**	
White	343
Non Hispanic or Latino	334
Hispanic or Latino	5
Not further specified	4
Asian	5
Non-White, non-Asian	8
**Age at original diagnosis (years), mean, median, (range) 56, 55 (15–87)**	
≤ 30	24
31–40	34
41–50	61
51–60	83
61–70	64
71–80	64
≥81	19
Age unknown	7
**Incidence of oncogenic** ***BRAF600/601***, ***RAS12/13/16**,* **and any** ***NF1*** **mutations**	
*BRAF* 600,601 codon mutations	156
*V600E*	127
*V600K*	22
*V600R*	3
*K601KE*	4
*RAS* mutations (canonical, any RAS type)	110
*NRASQ61*	95
*NRASG12, G13*	7
*KRASG12, G13, Q61*	6
*HRASG13, Q61*	2
*NF1* mutations (highly functional)	27
*Splice*	5
*Stop codon*	22
**DNA molecular classification for the current patients**	
*BRAF* codon 600/601 alone	157
*RAS* codon 12/13/61 alone	104
*NF1* alone (highly functional^*^)	21
*RAS* Codon 12/13/61 and NF1 (highly functional^*^)	6
*BRAF* codon 600/601 and RAS	1
Triple Wild type	67
**Gene expression classification**	
Immune-high	169
MITF-low	111
Keratin-high	76

* *The definition of highly functional somatic mutation is based on the computational analysis using the IMPACT assay*.

**Figure 3:**
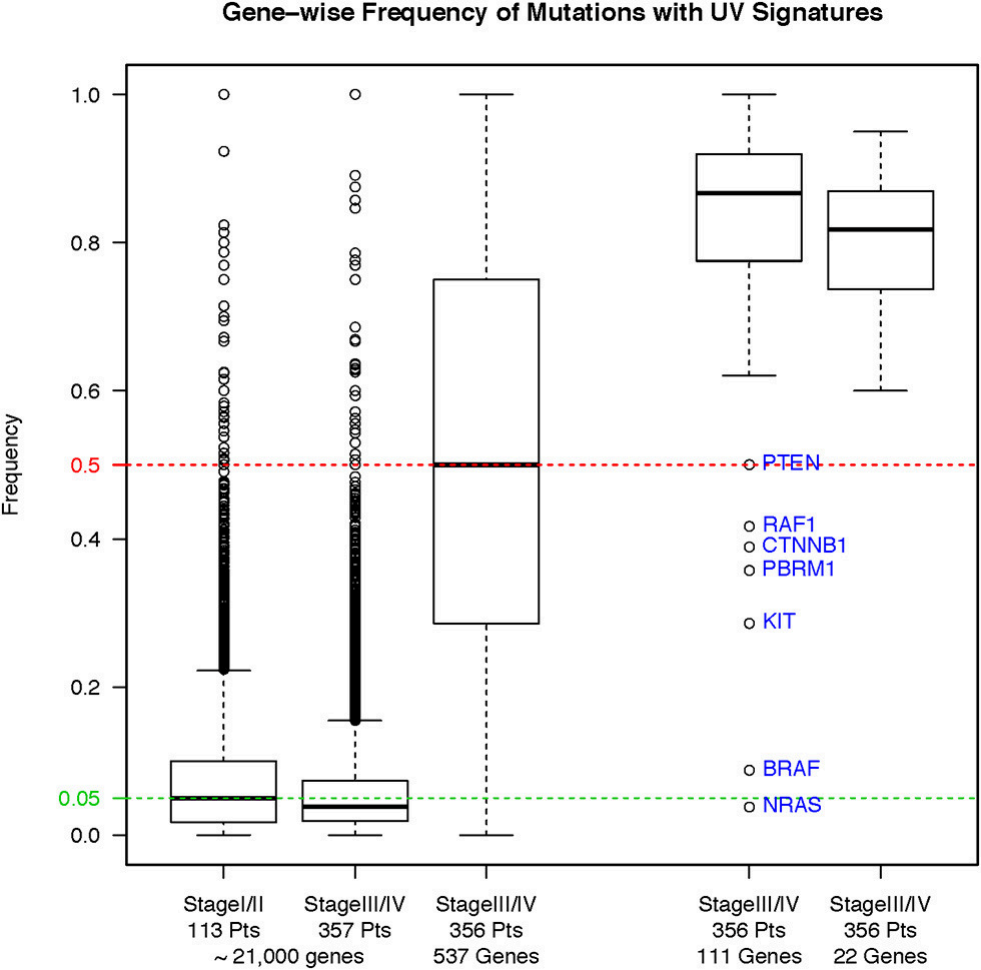
Frequency of ultraviolet-signature mutations across different sample subgroups before and after our filtering strategy. Results are shown as boxplots with median and 25/75 percentiles. Asterisks show significant differences at a *p*-value ≤ 0.05.

To assess whether our filtering strategy retained known somatic mutations, we tested the impact of this strategy on the hotspot mutations in five known cancer-associated genes: *BRAF, RAS* family (*HRAS, NRAS*, and *KRAS*), and stop-gain *NF1* gene mutations. We found only 4/303 total hotspot mutations fell outside the quality filtering criteria (Supplemental Material, Figure S2). We also compared the incidence of ultraviolet signature mutations in the unfiltered primary melanomas (*n* = 113), the unfiltered MM (*n* = 357), and the filtered MM (*n* = 356). Incidence of ultraviolet signature mutations (C>T substitutions) is significantly higher in unfiltered primary melanomas compared to MM samples (Figure 3, left panel, *p* < 2.2 × 10^−16^, Wilcoxon rank-sum test). Overall, C>T transitions, C>T transitions at a dipyrimidine site, and CC → TT accounted for 79.4%, 77.8%, and 0.11% of the total 5,322 mutations, respectively. Our filtering strategy found a significantly increased incidence of ultraviolet signature in retained vs. filtered mutations in the MM subgroup (*p* < 2.2 × 10^−16^, Wilcoxon). Nevertheless, mutations in other previously reported genes (e.g. *PTEN, RAF1, CTNNB1, PBRM1*, and *KIT*) were also retained (Figure 3, right panel). Thus, our filtering strategy retained high-quality mutation calls within known oncogenic driver genes.

Table S1 shows that 128 (OS-original diagnosis analysis) and 133 genes (OS-specimen collection analysis) were mutated in >11 patients (3%). To further limit the number of multiple comparisons testing for OS analysis, we conventionally used the survival status at the time of last follow-up. Although survival status (dead vs. alive) is inherently dependent on the duration of follow-up, the majority of surviving subjects (*n* = 171) had sufficient follow-up; only 7 (4%) vs. 33 (19%) of the censored subjects had follow-up for < 1-year vs. < 2-years, respectively. Of these, 53 (OS-original diagnosis analysis) and 57 (OS-specimen collection analysis) genes were mutated ≥1.5 times or ≤ 0.67 times in deceased subjects compared to living subjects. Gene lists from each of the two OS analyses were similar. Although *BRAF, NRAS, NF1, TP53, PTEN, PPP6C, CTNNB1, PDGFRA, IDH1*, and *KIT* were significantly mutated, they did not exhibit >50% change in deceased subjects compared to living subjects. Additionally, no change was seen when analysis was restricted to hotspot mutations previously reported for *BRAF, NRAS, NF1, TP53, PTEN, ATM, CTNNB1*, and *PPP6C* genes (25).

### Cox Analysis of Somatically Mutated Genes in MM TCGA SKCM Samples

**Figure 4:**
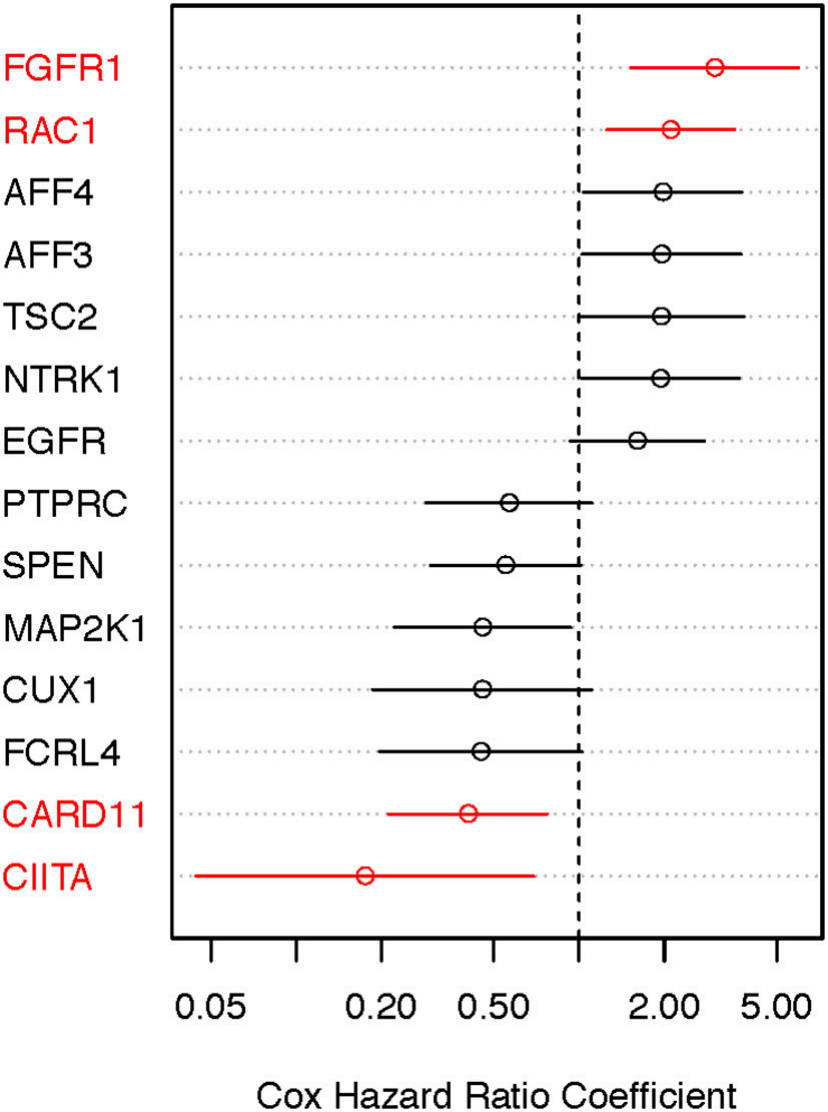
Identifying prognostically significant mutated genes using univariate Cox regression analysis (OS-original diagnosis) in the TCGA stage III/IV cohort. Following identification of clinically significant, high-quality mutations, genes bearing somatic mutations in more than 3% of cases were selected for age-adjusted univariate Cox analysis of OS. Hazard ratio [exp(coef)] point estimates with 95% confidence intervals for genes using the false discovery cutoff of 0.2 (red) or 0.2–0.4 (black) are shown.

**Figure 5:**
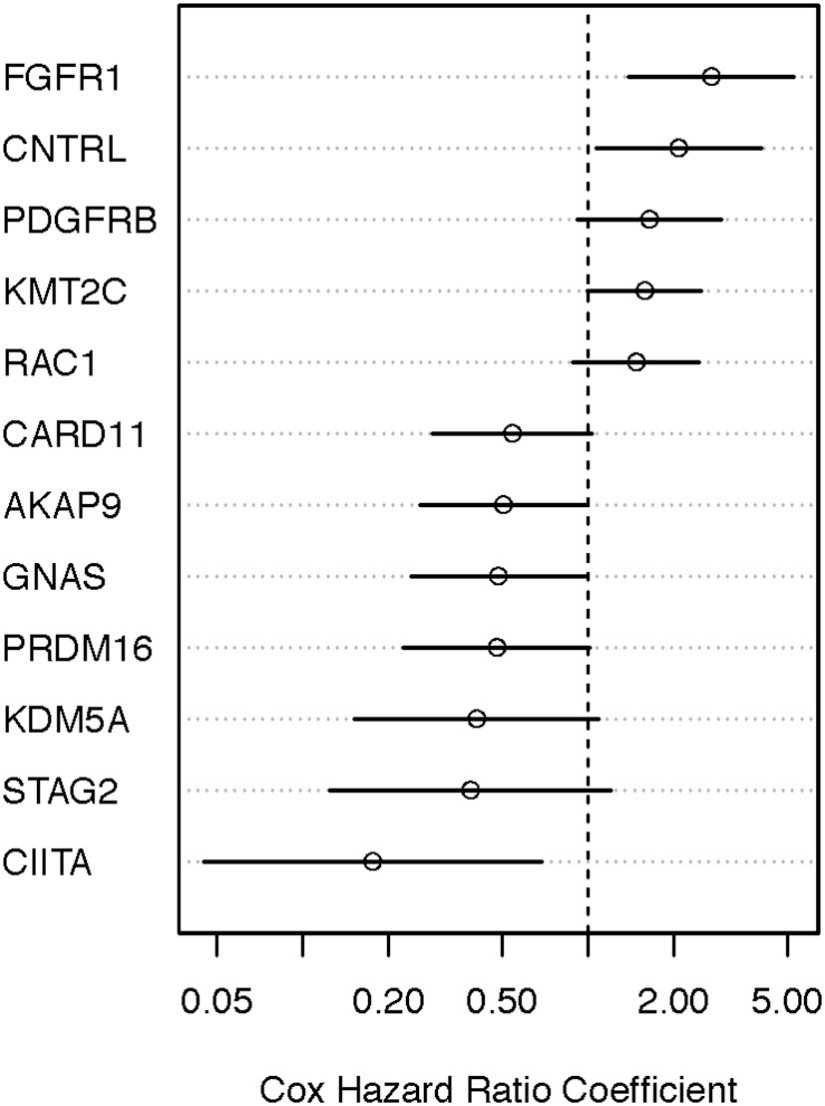
Identifying prognostically significant mutated genes using univariate Cox regression analysis (OS-specimen collection diagnosis) in the TCGA stage III/IV cohort. Following identification of clinically significant, high-quality mutations, genes bearing somatic mutations in more than 3% of cases were selected for age-adjusted univariate Cox analysis of OS. Hazard ratio [exp(coef)] point estimates with 95% confidence intervals for genes using the false discovery cutoff of 0.2 (red) or 0.2–0.4 (black) are shown.

We performed the age-adjusted univariate Cox regression analysis for each of the 53 genes (Supplemental Material, Table S1, panel A; OS-original diagnosis analysis). Using a FDR cutoff of 0.2, we found that 4/53 genes had prognostic potential: fibroblast growth factor receptor 1 (*FGFR1*), RAS-related C3 botulinum toxin substrate 1 (*RAC1*), caspase recruitment domain-containing protein 11 (*CARD11*), and class II major histocompatibility complex transactivator (*CIITA*). An additional 10 genes had less significance (FDR 0.2–0.4). When OS time from specimen collection was used for Cox analysis, 26 genes looked promising (FDR < 0.4). With the exception of *NTRK1, CUX1, TSC2*, and *AFF3*, all 10 genes that were identified in the OS-original diagnosis analysis were also found to have considerable prognostic significance (FDR < 0.4) in the OS-specimen collection analysis. Eight additional genes (*CNTRL, GNAS, AKAP9, KMT2C, PRDM16, KDM5A, PDGFRB*, and *STAG2*) also had prognostic value (FDR < 0.4, Cox *p*-value ≤ 0.10). Figures 4, 5 show hazard ratio point estimates with 95CI for these genes that were identified out of the OS from original diagnosis and OS from time to specimen collection analyses, respectively. The previously reported *CDKN2A, ARID2*, and *FBXW7* genes (26) exhibit at least 50% (fold change ≥1.5/ ≤ 0.67) difference in deceased subjects vs. living subjects. However, none of these genes met the FDR cutoff, even when analysis was focused on hotspot mutations (25).

### Properties of the Somatic Mutations in the 22 Genes in MM Samples

Twenty-two genes were mutated in 190 (53.4%) specimens. These genes include the 14 identified to be promising for prognostic significance from the OS analysis from original diagnosis of melanoma (Figure 4) plus the eight genes that were uniquely found to be promising for prognostic significance from the OS analysis from time of specimen collection, since *CIITA, RAC1, CARD11*, and *FGFR1* were common in both datasets (Figure 5). As shown in Figure 3, right panel, the majority of the mutations bear the ultraviolet signature (median frequency, 83.3%, ~95CI 78.6–88.0%). There was significant difference in the frequency of ultraviolet signature mutations seen in the 22 genes compared to the remaining 111 genes that were mutated in ≥3% of patients in either OS analyses (*p* = 0.04, Wilcoxon). Compared with the MAF of *BRAFV600* mutations, the MAFs for most genes from the 22-gene list were significantly lower (Supplemental Material, Figure S2, panel A). However, while the MAF of *BRAFV600* mutations was higher in primary as opposed to MM samples, the corresponding MAF for most of the 22 genes was the opposite—namely, higher in MM compared to primary melanoma specimens (Supplemental Material, Figure S3, panel B). The higher frequency of *BRAFV600* mutations in SKCM suggests their founder status in this disease, while supporting the notion that mutations in the other 22 genes are potentially clonal or subclonal events that occur more frequently in MM samples.

**Figure 6:**
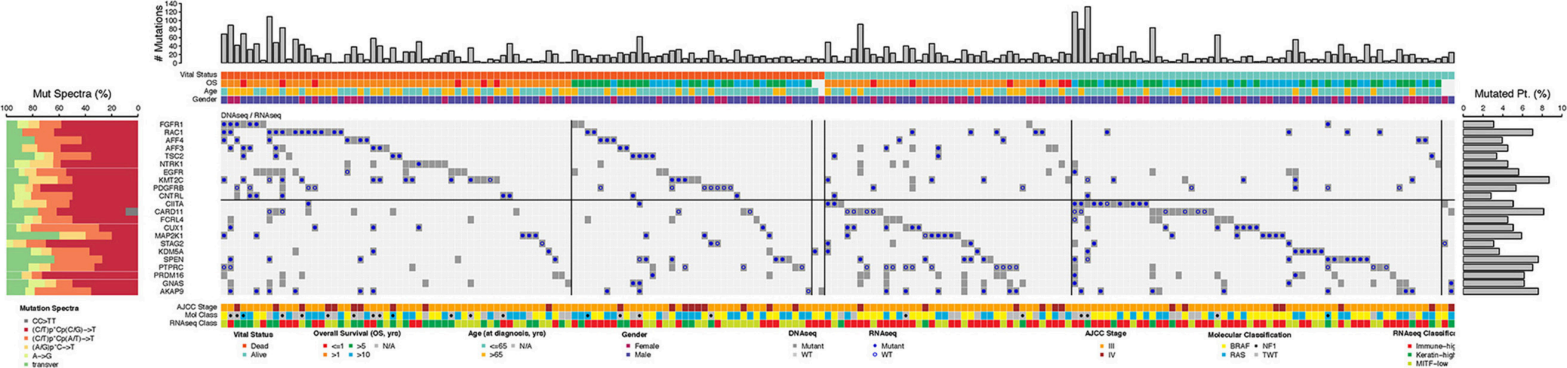
Landscape of somatically mutated genes that are promising for being prognostic (*n* = 22, FDR < 0.4) in the TCGA stage III/IV cohort following OS-original diagnosis and OS-time from specimen collection analyses. Only tumors with mutations in at least one of the 22 genes are shown in the center of the matrix (190 out of a total of 356). Mutated genes are shown as a binary outcome (presence/absence) for each sample and are color-coded according to the presence/absence of mutations as identified by DNA-seq (dark gray or light gray) and RNA-seq analysis [filled circles (∙) or open circles (°)]. Total number of somatic mutations (# Mutations, top), vital status, age at specimen collection (≤ 65, >65 years old), OS follow-up (in years), AJCC stage at specimen procurement, molecular classification [*BRAF* hotspot, *RAS* hotspot, *NF1* mutations (□ indicates nonsense mutation), triple wild-type (TWT)], RNA-seq classification, mutation spectra (left panel), and mutated gene frequency (right panel) for each of the 14 mutated genes (left) are shown for each sample.

Figure 6 shows the landscape of the 22 genes that demonstrated prognostic significance in the 190 specimens. As expected, vital status was not associated with total somatic mutation burden, but was associated with “immune-high” RNA-seq signatures (*p* = 0.873 and *p* = 1.78 × 10^−7^, respectively; Chi-square test). Overall, the frequency of mutations in any of these 22 genes was < 10% in the MM samples. Mutated *RAC1, FGFR1, AFF3*, and *NTRK1* genes were more frequently seen in MM samples from patients who died within 5 years from the original diagnosis. In contrast, mutated *CIITA* was more frequently seen in MM samples from patients who have lived >5 years after original diagnosis (Figure 6). All 22 genes, except *MAP2K1*, were significantly associated with high somatic mutation burden (FDR = 0.09 for *MAP2K1*, FDR < 0.001 for the rest). Somatic mutations in the haploinsufficient homeodomain transcription factor gene, cut-like homeobox 1 (*CUX1*) (27), were differentially distributed in the “immune-high” vs. non “immune-high” specimens (*p* = 0.027).

UNCeqR analysis revealed that 271 of these 423 mutation events (64.1%) were sufficiently covered with the exception of *NTRK1, EGFR*, and *GNAS* (6, 10, and 23%, respectively), implying low-to-absent gene expression (Figure 6). Of the remaining 19 genes, somatic mutations in only 9 genes were confirmed by the RNA-seq data in more than 75% of samples (*RAC1, AFF4, TSC2, CNTRL, CUX1 MAP2K1, KDM5A, SPEN, AKAP9*), although corresponding genes were expressed based on the RNA-Seq by Expectation-Maximization (RSEM) data. Figure S4 (Supplemental Material) further characterizes somatic mutations in these 9 genes that were confirmed by RNA-seq in stage III/IV SKCM.

### Validation of the 22 Genes in an Independent Cohort

To validate our findings related to the 22 genes from the TCGA cohort in a separate MM cohort, we analyzed the presence of these mutations in a cohort of 33 stage III/IV patients with SKCM who were followed at the UNC-CH Melanoma Program and had consented to the UNCseq™ project (Supplemental Material, Table S2), combined with 6 other previously published melanoma datasets (18–20, 22, 26, 28). Table 2 shows the non-synonymous somatic mutations from MM tumor samples of cutaneous or unknown primaries that were subjected to next generation sequencing analysis and grouped according to primary (*n* = 107) vs. metastatic (*n* = 417) status. As was the case with the TCGA SKCM cohort, the frequency for the 22 somatically mutated genes in MM was low, with few exceptions (*KMT2C, PRDM16, CARD11, PTPRC*). In contrast with the TCGA SKCM cohort, however, more mutated genes were seen in primaries compared to metastases (2/22 in the TCGA cohort vs. 10/22 in the validation cohort). Of the 9 mutated genes whose mutations were confirmed by RNA-seq, all but one (i.e., AFF4) were equivocally found to be expressed in the other two datasets that reported both RNA and DNA sequencing analysis (19, 26).

**Table 2:** Frequency of nonsynonymous somatic mutations in the validation (non-TCGA) cohort for the 22 genes that were considered promising for prognostic significance in melanoma from the TCGA cohort analysis.

	**Primary Cutaneous Melanoma Samples**				**Metastatic Cutaneous or Unknown Primary Melanoma Tumor Samples**							
	**Australia**	**Yale**	**Hodis**	**Total (%)**	**Hodis**	**Australia**	**Yale**	**MSKCC**	**UCLA**	**UNC-CH**	**DFCI/Broad**	**Total (%)**
	**(*n* = 68)**	**(*n* = 20)**	***(n* = 15)**	**(*n* = 103)**	***(n* = 30)**	**(*n* = 92)**	**(*n* = 7)**	**(*n* = 61)**	***(n* = 38)**	**(*n* = 33)**	**(*n* = 25)**	**(*n* = 349)**
RAC1	5	2	0	7 (7)	2+	3	5+	N/S	2	N/S	0	12 (5)*
AFF3	8	1	5	14 (14)	5-	5	3+	N/S	2	N/S	1	16 (6)*
PTPRC	12	7	2	21 (20)	7+	10	9-	N/S	5	N/S	2	33 (13)*
FCRL4	4	2	0	6 (6)	3-	3	6-	N/S	3	N/S	0	15 (6)*
NTRK1	3	2	1	6 (6)	3+	5	3-	2	2	4	0	19 (5)
CARD11	9	1	3	13 (13)	11-	5	11-	6	4	N/S	3	40 (13)*
CIITA	4	0	2	6 (6)	2+	2	3-	N/S	0	N/S	1	8 (3)*
MAP2K1	3	0	1	4 (4)	3+	2	2+	2	2	3	1	15 (4)
SPEN	4	4	0	8 (8)	5+	9	8+	N/S	3	5	2	32 (11)*
EGFR	5	2	4	11 (11)	4+	10	8-	2	1	3	0	28 (8)
FGFR1	2	2	2	6 (6)	2-	3	1+	1	2	1	0	10 (3)
CUX1	5	4	6	15 (15)	3+	8	7+	N/S	4	N/S	1	23 (9)*
AFF4	1	1	2	4 (4)	2-	5	1+	N/S	1	N/S	1	10 (4)*
TSC2	3	1	0	4 (4)	6+	5	4+	1	3	0	3	22 (6)
CNTRL	1	3	0	4 (4)	0	3	2+	N/S	2	N/S	0	7 (3)*
AKAP9	5	4	1	10 (10)	4+	4	14+	N/S	2	N/S	3	27 (11)*
GNAS	6	2	0	8 (8)	2+	10	7+	4	3	7	1	34 (10)
KMT2C	7	4	11	22 (21)	14-	14	8+	13	5	N/S	11	65 (21)*
PRDM16	7	3	6	16 (16)	5+	12	11-	N/S	3	N/S	4	35 (14)*
KDM5A	2	0	1	3 (3)	6-	1	3+	N/S	3	N/S	4	17 (7)*
PDGFRB	3	1	2	6 (6)	3–	2	8-	2	2	2	1	20 (8)
STAG2	1	2	0	3 (3)	4+	3	3+	1	0	N/S	1	12 (4)*
UV+/mut+	31	NR	NR	31 (46)	NR	51	NR	NR	NR	NR	NR	51 (55)
UV–/mut+	2			2 (3)		3						2 (3)
UV+/mut–	21			21 (31)		22						21 (24)
UV–/mut–	14			14 (21)		16						16 (17)

Only next generation sequencing data from tumor samples of cutaneous or unknown primary that were procured before systemic treatment and had matched normal tissue samples are shown. Two studies [UNC-CH and (22)] reported on targeted panel sequencing; therefore, the mutation status for several genes of interest was not studied (N/S) across all genes (*). Incidence of somatic mutations in primary vs. metastatic melanoma samples are shown separately. Cases with non-synonymous somatic mutations are shown for each dataset and for the entire cohort. Two studies (19, 26) reported whether mutations were identified at the transcript level (+) or not (–). A single study (21) reported on whether tumor samples had evidence of UV signature mutations. Patient follow-up and status (alive or dead) was reported on 4 studies (highlighted in brown).

**Figure 7:**
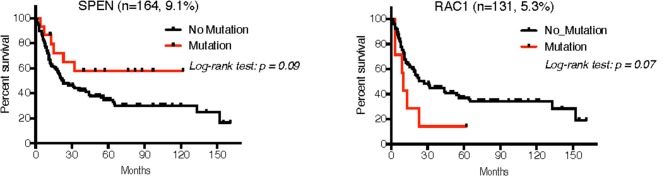
Overall survival analysis of patients with MM in the validation cohort that had follow-up according to the mutation status of *SPEN* and *RAC1* using the Kaplan-Meier method. Although 225 patients had available follow-up across 5 studies, only 3 studies had available mutation data for these two genes (18, 19, 21).

Of the seven studies that comprised our validation cohort, we were able to obtain reliable follow-up data from 224 patients across five studies (UNC-CH plus 18, 19, 21, 22. The median follow-up of this patient cohort was 20.0 months (~95CI, 16.9–27.0 months), during which time 30.4% of deaths occurred. Cox analysis for each of the 8 mutated genes whose RNA expression was confirmed across all studies showed trends of mutations in *RAC1* in MM with worse prognosis (HR = 2.1, 95CI 0.66–6.63, log-rank *p* = 0.07), whereas mutations in *SPEN* showed trends of mutations with better prognosis (HR = 0.50, range 0.27–0.93, log-rank *p* = 0.09) (Figure 7).

### Beyond *SPEN* and *RAC1* Somatic Mutations; Copy Number Alterations and Protein Expression

**Figure 8:**
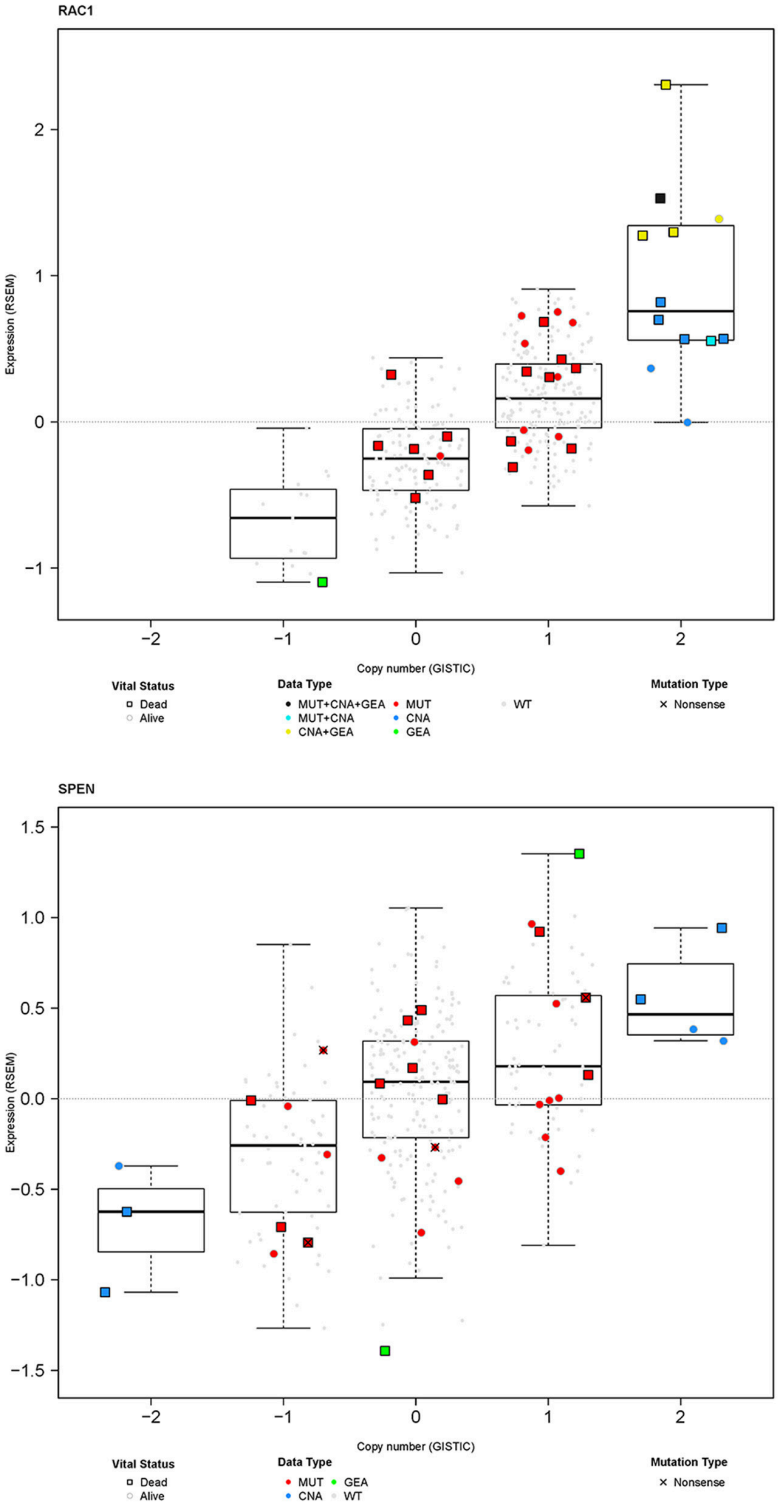
Integration of somatic mutations (red), gene copy number (blue), and gene expression (green) for *RAC1* and *SPEN* in the MM TCGA dataset. Results are shown as RNA expression (log_2_-transformed RSEM data) over copy number alterations that have been generated using the GISTIC algorithm. For better visualization, samples with no somatic mutations are shown as small gray dots or squares.

In general, mutations are frequently associated with copy number alterations and can influence gene expression (29–31). Furthermore, genetic aberrations may have an impact on the function and/or stability of RNA which may lead to corresponding changes in protein function and/or abundance. Figure 8 shows integrated analysis of somatic mutations, copy number alterations, and gene expression alterations for *RAC1* and *SPEN* for the 357 TCGA MM samples. All, but two, somatic mutations were not associated with definite copy number alterations (GISTIC score 2+ or 2–) and all, but one, somatic mutations were not associated with changes in gene expression. However, 22/34 patients with *any* genetic aberrations in *RAC1* (somatic mutations and/or copy number amplifications) were deceased at the time of analysis. The unfavorable outcome of patients with any genetic aberrations in *RAC1* is in line with a report on the adverse prognostic significance of high RAC1 protein expression by immunohistochemistry in primary cutaneous melanoma samples (32).

**Figure 9:**
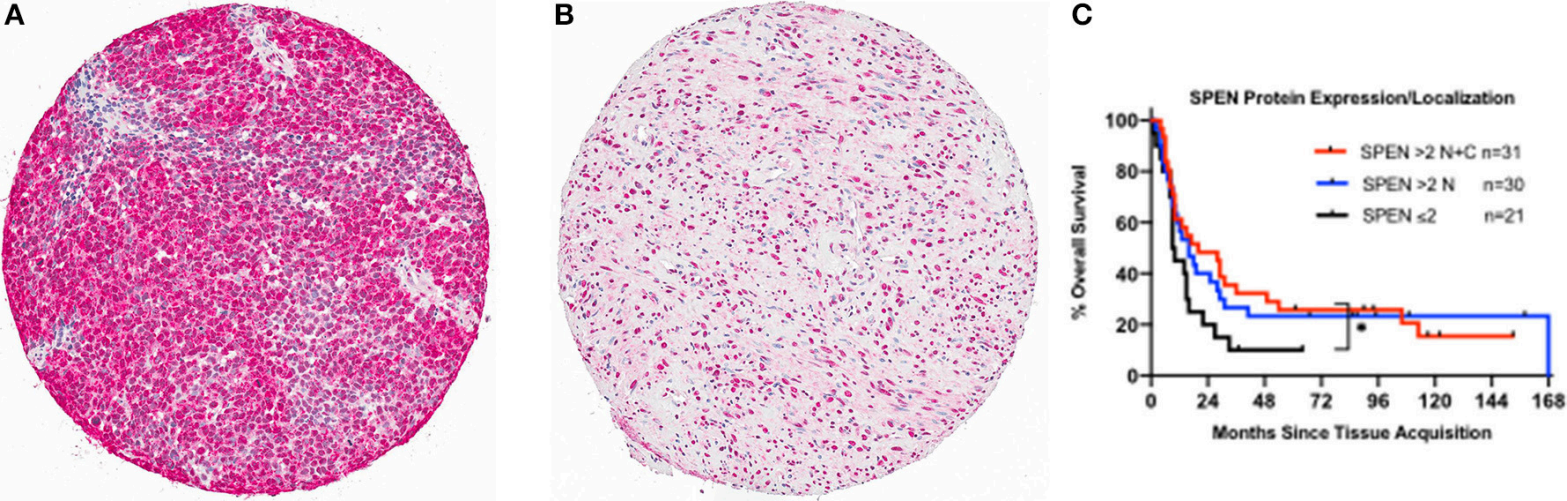
Protein expression of SPEN in MM. **(A,B)**. Images of representative tissue sections from the MM UNC-CH tissue microarray (10X magnification) that were stained with an anti-SPEN antibody (see materials and methods for details). Differences in SPEN staining intensity (**A**, 3+; **B**, 2+), expression of SPEN in melanoma vs. stromal cells **(B)**, and localization (**A**, nuclear plus cytoplasmic; **B**, nuclear only) are shown. **(C)**. Melanoma-specific OS analysis from time of specimen collection to the last follow-up in the 87 MM UNC-CH dataset. Of these patients only 82 had available melanoma-specific OS data.

Somatic mutations in *SPEN* were not associated with any definite copy number alterations (GISTIC score 2+ or 2–) or clear changes in gene expression. To investigate the protein expression pattern in SPEN, we performed immunohistochemical analysis in MM samples procured from 87 patients who were treated at UNC-CH Melanoma Program between 2000 and 2011. Clinicopathologic characteristics of this patient cohort are shown in Table S3. Protein expression of SPEN by melanoma cells was predominantly nuclear (76%); 23.5% of cores expressed both strong (2+ and 3+) nuclear and cytoplasmic stain (23.5%) and 0.5% of cores expressed predominantly cytoplasmic stain (Figures 9A,B). SPEN expression was overall higher by melanoma cells compared to stromal cells (Figure 9B). Using the semiquantitative 0, 1+, 2+, 3+ scale, 0%, 9.3%, 28.3%, 62.3% of tissue cores exhibited absent, low, intermediate, or strong expression of SPEN by melanoma cells. Strong expression staining intensity (2+ and 3+) for SPEN protein, if present in both nuclear and cytoplasmic compartments in melanoma cells, trended to correlate with the presence of tumor-infiltrating lymphocytes (2-way contingency table, χ^2^ test *p* = 0.08). At a median follow-up of 15 months (range 1–68 months) and for the patients who had melanoma-specific overall survival data (*n* = 82) patients with high (>2+) nuclear plus cytoplasmic expression of SPEN by melanoma cells had longer melanoma-specific OS compared with patients with low expression (≤ 2+) (HR and 95CI, 0.56, 0.29–1.09, log-rank *p*-value 0.048) (Figure 9C). No significant differences were observed between the other group comparisons. Given that *SPEN* somatic mutations do not associate with significant differences in RNA expression, we speculate that distinct yet-to-be identified *SPEN* mutations may regulate SPEN localization.

## Discussion

We have analyzed the largest to-date sample cohort for somatic mutations in MM. With available follow-up information from the TCGA, we validated our findings in an independent dataset. In contrast to previous work that defined the landscape of somatic mutations in melanoma irrespective of stage and subtype (8, 19–21, 26, 28), our focus on MM was unique in that it attempted to understand the prognostic significance of mutations in established metastases specifically. We have selected for high-quality, functional, statistically significant mutations. The large number of tissue specimens, the focus on MM samples and somatic mutations only, and our filtering strategy are a few of the critical factors that differentiate our study results from previous efforts to identify prognostic factors in melanoma using a combination of clinicopathologic and multiple—omics platforms (33, 34). Our study has several important conclusions. Only a handful of genes that had been previously described as recurrently mutated may have prognostic significance (e.g., *RAC1*). Other well-characterized and more frequently mutated genes (e.g., *BRAF, RAS, NF1, TP53, PTEN, CDKN2A*) were not prognostic in MM, perhaps because such mutations are already found in nevi and primary melanomas (1). Less well-known genes in melanoma biology not only may be significantly mutated due to the random effect of ultraviolet radiation, but their mutated status may have potential prognostic significance [e.g., the *Spen homolog transcriptional regulator* (*SPEN*)]. Not all somatic mutations are expressed at the RNA level. Validation of expressed somatic mutations detected at the DNA level by RNA sequencing maybe the future in clinical oncology; current commercially available molecular tests that perform targeted panel sequencing identify mutations at the DNA level only (e.g., Foundation Medicine).

To date, there is limited knowledge about the significance of low-frequency somatic mutations in melanoma. Studies have established that ultraviolet radiation induces mutations, which in turn may potentially yield neoantigens. The neoantigens provoke an immune response, which may account for the immunogenicity of cutaneous melanoma and other cancers (35). The majority of the 22 genes that were identified from the TCGA and validation cohort were infrequently mutated, more frequently found in specimens with high somatic mutation burden, are evenly distributed across the entire gene. Also, they are of the missense type (except for *RAC1* and perhaps *MAP2K1*). The lack of mutations with high functional impact does not mitigate the importance of such missense mutations in protein function, as in the case of *KEAP1* in lung cancer (36). Instead, it may provide an explanation about the lack of association between high somatic mutation burden with host immune response and OS in melanoma, which is complex: somatic mutations in genes associated with immune surveillance (e.g., *PTPRC*/*CD45, FCRL4, CARD11)* may be associated with favorable prognosis because potentially damaging mutations are not ultimately expressed. In contrast, somatic mutations in tumor-promoting genes (*FGFR1, RAC1, AFF3, AFF4, TSC2*) may be intuitively associated with unfavorable prognosis.

Our validation cohort was comprised of patient samples with significant heterogeneity with respect to selection for *BRAFV600* mutations and, systemic treatment type (i.e., FDA approved treatments before or after 2011), which may have influenced OS (37, 38), and the geopolitical origin of patients (39). Despite the challenges in data interpretation, we were able to confirm the low somatic mutation frequency and the UV signature type of most mutations. Use of different bioinformatics algorithms may have accounted for the discrepancy in validation of expressed mutations by RNA-seq between the TCGA dataset and others. Nevertheless, certain mutated genes were confirmed by RNA-seq across all datasets (e.g., *RAC1, MAP2K1, SPEN, CUX1, TSC2, CNTRL, AKAP9*, and *STAG2*) whereas others were found not to be expressed, irrespective of the RNA-seq validation algorithm used (e.g., *FCRL4, CARD11, PDGFRB*).

Our survival analysis of the validation cohort was significantly underpowered to confirm the prognostic significance of the eight mutated genes that were also found to be expressed. Furthermore, the validation cohort had shorter follow-up and less events. For example, assuming that a given gene whose incidence of mutation is 5.3% in the study population (e.g., NTRK1) and is associated with worse OS (HR = 1.93), the power to detect significant prognostic difference if *n* = 245 and 70% of patients had an event (e.g., death) is only 0.64. Nevertheless, we were able to show that *RAC1* and *SPEN* may be promising prognostic factors for OS in MM. *RAC1* been previously reported in SKCM and other datasets (8, 20) and was associated with melanoma progression, suppression of host immune response, and drug resistance (40). A recent study has shown that high expression of RAC1 protein in primary cutaneous melanoma samples was associated with thinner melanomas, *BRAFV600* mutation and with *RAC1* mutation (32). Little is known about the role of *SPEN*, a transcriptional regulator of NOTCH1 and hormone receptor signaling, in melanoma and other cancers (41). In our work we have shown that high (>2+) nuclear and cytoplasmic SPEN expression trends to associate with melanomas that have present tumor-infiltrating lymphocytes. This association may account for our finding regarding the favorable prognostic significance of patients with high melanoma nuclear and cytoplasmic SPEN expression compared to those with lower (≤ 2+) protein expression. The results are comparable with a recent report that investigated the prognostic significance of high vs. low SPEN RNA expression in luminal A breast cancer (24). Irrespective of the limitations of our validation cohort, our study suggests that the prognostic impact for most of these genes may be small. Rather, multiple genetic, and epigenetic aberrations within the cancer itself (42), the host (43), and the environment (44) may play an even larger role in established metastases as opposed to primary melanoma.

## Ethics Statement

This study was carried out in accordance with the guidelines and recommendations from the Genomic Data Commons (GDC) Data Portal of the Cancer Genome Atlas Project. GDC Data Portal is an interactive data system for researchers to search, download, upload, and analyze harmonized cancer genomic data sets, including TCGA.

## Author Contributions

GP, MH, AS, MP, DB, AE, JF, WH, JW, CQ, and SM involved in data collection. XZ, Al, PL, AH, JP, DM, MS, HJ, SJ, KH, DO, NT, PG, FC, CL, HE, NS, WH, JW, CQ, NW, PJ, JT, NH, GM, RL, DJ, RS, DH, and SM involved in data analysis. XZ, GP, KH, CQ, RS, DH, and SM involved in manuscript preparation.

### Conflict of Interest Statement

The authors declare that the research was conducted in the absence of any commercial or financial relationships that could be construed as a potential conflict of interest.

## Abbreviations

SKCM: Skin Cutaneous Melanoma
MM: metastatic melanoma
TCGA: The Cancer Genome Atlas Project
VCF: variant call format
UNC-CH: the University of North Carolina at Chapel Hill
OS: overall survival
RNA-seq: RNA sequencing
95CI: 95% confidence intervals
FDR: false discovery rate
MAF: mutant allele frequency
MAC: mutant allele count.

## References

[1] Shain AH Yeh I Kovalyshyn I Sriharan A Talevich E Gagnon A. The genetic evolution of melanoma from precursor lesions. N Engl J Med. (2015) 373:1926–36. 10.1056/NEJMoa1502583 26559571

[2] Thomas NE Edmiston SN Alexander A Groben PA Parrish E Kricker A Association between NRAS and BRAF mutational status and melanoma-specific survival among patients with higher-risk primary melanoma. JAMA Oncol. (2015) 1:359–68. 10.1001/jamaoncol.2015.0493 26146664 PMC4486299

[3] Hosler GA Davoli T Mender I Litzner B Choi J Kapur P. A primary melanoma and its asynchronous metastasis highlight the role of BRAF, CDKN2A, and TERT. J Cutan Pathol. (2015) 42:108–17. 10.1111/cup.12444 25407517 PMC4470704

[4] Nagore E Heidenreich B Rachakonda S Garcia-Casado Z Requena C Soriano V. TERT promoter mutations in melanoma survival. Int J Cancer (2016) 139:75–84. 10.1002/ijc.30042 26875008 PMC8238633

[5] Bai X Kong Y Chi Z Sheng X Cui C Wang X. MAPK pathway and TERT promoter gene mutation pattern and its prognostic value in melanoma patients: a retrospective study of 2,793 cases. Clin Cancer Res. (2017) 23:6120–27. 10.1158/1078-0432.CCR-17-0980 28720667

[6] Griewank KG Murali R Puig-Butille JA Schilling B Livingstone E Potrony M. TERT promoter mutation status as an independent prognostic factor in cutaneous melanoma. J Natl Cancer Inst. (2014) 106:dju246. 10.1093/jnci/dju246 25217772 PMC4200061

[7] Roh MR Gupta S Park KH Chung KY Lauss M Flaherty KT. Promoter methylation of PTEN is a significant prognostic factor in melanoma survival. J Invest Dermatol. (2016) 136:1002–11. 10.1016/j.jid.2016.01.024 26854490

[8] Cancer Genome Atlas N Genomic Classification of Cutaneous Melanoma. Cell (2015) 161:1681–96. 10.1016/j.cell.2015.05.044 26091043 PMC4580370

[9] Jakob JA Bassett RL Ng CS Curry JL Joseph RW Alvarado GC. NRAS mutation status is an independent prognostic factor in metastatic melanoma. Cancer (2012) 118:4014–23. 10.1002/cncr.26724 22180178 PMC3310961

[10] Long GV Menzies AM Nagrial AM Haydu LE Hamilton AL Mann GJ. Prognostic and clinicopathologic associations of oncogenic BRAF in metastatic melanoma. J Clin Oncol. (2011) 29:1239–46. 10.1200/JCO.2010.32.4327 21343559

[11] Frampton GM Fichtenholtz A Otto GA Wang K Downing SR He J. Development and validation of a clinical cancer genomic profiling test based on massively parallel DNA sequencing. Nat Biotechnol. (2013) 31:1023–31. 10.1038/nbt.2696 24142049 PMC5710001

[12] Conley BA Doroshow JH. Molecular analysis for therapy choice: NCI MATCH. Semin Oncol. (2014) 41:297–9. 10.1053/j.seminoncol.2014.05.002 25023344

[13] Schlenk RF Döhner K Krauter J Fröhling S Corbacioglu A Bullinger L. Mutations and treatment outcome in cytogenetically normal acute myeloid leukemia. N Engl J Med. (2008) 358:1909–18. 10.1056/NEJMoa074306 18450602

[14] Sanborn JZ Chung J Purdom E Wang NJ Kakavand H Wilmott JS. Phylogenetic analyses of melanoma reveal complex patterns of metastatic dissemination. Proc Natl Acad Sci USA. (2015) 112:10995–10000. 10.1073/pnas.1508074112 26286987 PMC4568214

[15] Harbst K Lauss M Cirenajwis H Isaksson K Rosengren F Törngren T. Multiregion whole-exome sequencing uncovers the genetic evolution and mutational heterogeneity of early-stage metastatic melanoma. Cancer Res. (2016) 76:4765–74. 10.1158/0008-5472.CAN-15-3476 27216186

[16] Cingolani P Platts A Wang le L Coon M Nguyen T Wang L. A program for annotating and predicting the effects of single nucleotide polymorphisms, SnpEff: SNPs in the genome of drosophila melanogaster strain w1118; iso-2; iso-3. Fly (2012) 6:80–92. 10.4161/fly.19695 22728672 PMC3679285

[17] Wilkerson MD Cabanski CR Sun W Hoadley KA Walter V Mose LE. Integrated RNA and DNA sequencing improves mutation detection in low purity tumors. Nucleic Acids Res. (2014) 42:e107. 10.1093/nar/gku489 24970867 PMC4117748

[18] Hugo W Zaretsky JM Sun L Song C Moreno BH Hu-Lieskovan S. Genomic and transcriptomic features of response to anti-PD-1 therapy in metastatic melanoma. Cell (2016) 165:35–44. 10.1016/j.cell.2016.02.065 26997480 PMC4808437

[19] Krauthammer M Kong Y Bacchiocchi A Evans P Pornputtapong N Wu C. Exome sequencing identifies recurrent mutations in NF1 and RASopathy genes in sun-exposed melanomas. Nat Genet. (2015) 47:996–1002. 10.1038/ng.3361 26214590 PMC4916843

[20] Krauthammer M Kong Y Ha BH Evans P Bacchiocchi A McCusker JP. Exome sequencing identifies recurrent somatic RAC1 mutations in melanoma. Nat Genet. (2012) 44:1006–14. 10.1038/ng.2359 22842228 PMC3432702

[21] Hayward NK Wilmott JS Waddell N Johansson PA Field MA Nones K. Whole-genome landscapes of major melanoma subtypes. Nature (2017) 545:175–80. 10.1038/nature22071 28467829

[22] Catalanotti F Cheng DT Shoushtari AN Johnson DB Panageas KS Momtaz P PTEN loss-of-function alterations are associated with intrinsic resistance to BRAF inhibitors in metastatic melanoma. JCO Precis Oncol. (2017). 10.1200/PO.16.00054 PMC7446400 32913971

[23] Zhao X Wang A Walter V Patel NM Eberhard DA Hayward MC. Combined targeted DNA sequencing in non-small cell lung cancer (NSCLC) using UNCseq and NGScopy, and RNA sequencing using UNCqeR for the detection of genetic aberrations in NSCLC. PLoS ONE (2015) 10:e0129280. 10.1371/journal.pone.0129280 26076459 PMC4468211

[24] Légaré S Cavallone L Mamo A Chabot C Sirois I Magliocco A. The estrogen receptor cofactor SPEN functions as a tumor suppressor and candidate biomarker of drug responsiveness in hormone-dependent breast cancers. Cancer Res. (2015) 75:4351–63. 10.1158/0008-5472.CAN-14-3475 26297734

[25] Chang MT Asthana S Gao SP Lee BH Chapman JS Kandoth C. Identifying recurrent mutations in cancer reveals widespread lineage diversity and mutational specificity. Nat Biotechnol. (2016) 34:155–63. 10.1038/nbt.3391 26619011 PMC4744099

[26] Hodis E Watson IR Kryukov GV Arold ST Imielinski M Theurillat JP. A landscape of driver mutations in melanoma. Cell (2012) 150:251–63. 10.1016/j.cell.2012.06.024 22817889 PMC3600117

[27] Wong CC Martincorena I Rust AG Rashid M Alifrangis C Alexandrov LB. Inactivating CUX1 mutations promote tumorigenesis. Nat Genet. (2014) 46:33–8. 10.1038/ng.2846 24316979 PMC3874239

[28] Berger MF Hodis E Heffernan TP Deribe YL Lawrence MS Protopopov A. Melanoma genome sequencing reveals frequent PREX2 mutations. Nature (2012) 485:502–6. 10.1038/nature11071 22622578 PMC3367798

[29] Ding J McConechy MK Horlings HM Ha G Chun Chan F Funnell T. Systematic analysis of somatic mutations impacting gene expression in 12 tumour types. Nat Commun. (2015) 6:8554. 10.1038/ncomms9554 26436532 PMC4600750

[30] Zack TI Schumacher SE Carter SL Cherniack AD Saksena G Tabak B. Pan-cancer patterns of somatic copy number alteration. Nat Genet. (2013) 45:1134–40. 10.1038/ng.2760 24071852 PMC3966983

[31] Roszik J Wu CJ Siroy AE Lazar AJ Davies MA Woodman SE. Somatic copy number alterations at oncogenic loci show diverse correlations with gene expression. Sci Rep. (2016) 6:19649. 10.1038/srep19649 26787600 PMC4726397

[32] Mar VJ Wong SQ Logan A Nguyen T Cebon J Kelly JW. Clinical and pathological associations of the activating RAC1 P29S mutation in primary cutaneous melanoma. Pigment Cell Melanoma Res. (2014) 27:1117–25. 10.1111/pcmr.12295 25043693

[33] Jiang Y Shi X Zhao Q Krauthammer M Rothberg BE Ma S. Integrated analysis of multidimensional omics data on cutaneous melanoma prognosis. Genomics (2016) 107:223–30. 10.1016/j.ygeno.2016.04.005 27141884 PMC4893887

[34] Jayawardana K Schramm SJ Haydu L Thompson JF Scolyer RA Mann GJ. Determination of prognosis in metastatic melanoma through integration of clinico-pathologic, mutation, mRNA, microRNA, and protein information. Int J Cancer (2015) 136:863–74. 10.1002/ijc.29047 24975271

[35] Brown SD Warren RL Gibb EA Martin SD Spinelli JJ Nelson BH. Neo-antigens predicted by tumor genome meta-analysis correlate with increased patient survival. Genome Res. (2014) 24:743–50. 10.1101/gr.165985.113 24782321 PMC4009604

[36] Hast BE Cloer EW Goldfarb D Li H Siesser PF Yan F Cancer-derived mutations in KEAP1 impair NRF2 degradation but not ubiquitination. Cancer Res. (2014) 74:808–17. 10.1158/0008-5472.CAN-13-1655 24322982 PMC3932503

[37] Wolchok JD Chiarion-Sileni V Gonzalez R Rutkowski P Grob JJ Cowey CL. Overall survival with combined nivolumab and ipilimumab in advanced melanoma. N Engl J Med. (2017) 377:1345–56. 10.1056/NEJMoa1709684 28889792 PMC5706778

[38] Long GV Eroglu Z Infante J Patel S Daud A Johnson DB. Long-term outcomes in patients with BRAF V600-mutant metastatic melanoma who received dabrafenib combined with trametinib. J Clin Oncol. (2018) 36:667–73. 10.1200/JCO.2017.74.1025 28991513 PMC10466457

[39] Karimkhani C Green AC Nijsten T Weinstock MA Dellavalle RP Naghavi M. The global burden of melanoma: results from the global burden of disease study 2015. Br J Dermatol. (2017) 177:134–40. 10.1111/bjd.15510 28369739 PMC5575560

[40] Watson IR Li L Cabeceiras PK Mahdavi M Gutschner T Genovese G. The RAC1 P29S hotspot mutation in melanoma confers resistance to pharmacological inhibition of RAF. Cancer Res. (2014) 74:4845–52. 10.1158/0008-5472.CAN-14-1232-T 25056119 PMC4167745

[41] Stephens PJ Davies HR Mitani Y Van Loo P Shlien A Tarpey PS. Whole exome sequencing of adenoid cystic carcinoma. J Clin Invest. (2013) 123:2965–8. 10.1172/JCI67201 23778141 PMC3999050

[42] Mo Q Wang S Seshan VE Olshen AB Schultz N Sander C. Pattern discovery and cancer gene identification in integrated cancer genomic data. Proc Natl Acad Sci USA. (2013) 110:4245–50. 10.1073/pnas.1208949110 23431203 PMC3600490

[43] Blank CU Haanen JB Ribas A Schumacher TN. CANCER IMMUNOLOGY. The “cancer immunogram”. Science (2016) 352:658–60. 10.1126/science.aaf2834 27151852

[44] Zitvogel L Daillère R Roberti MP Routy B Kroemer G. Anticancer effects of the microbiome and its products. Nat Rev Microbiol. (2017) 15:465–78. 10.1038/nrmicro.2017.44 28529325

