# Supplementary material for: The Prognostic Significance of Low-Frequency Somatic Mutations in Metastatic Cutaneous Melanoma
Source: Front Oncol. 2019 Jan 4;8:584. doi: 10.3389/fonc.2018.00584 (PMC6329304; doi:10.3389/fonc.2018.00584)
Supplement: Supplementary file 1 [file Data_Sheet_1.docx]

**Supplemental Material on *“The Prognostic Significance of Low-Frequency Somatic Genetic Aberrations in Metastatic Cutaneous Melanoma”***

**Table of Contents**

**Supplement on Patients and Methods …………………………………………………………... 2**

**Supplement on Results …………………………………………………………………………… 3**

**Supplementary Figures …………………………………………………………………………... 4**

**Supplementary Tables …………………………………………………………………………… 9**

**References on Data Supplement ………………………………………………………………… 15**

**Supplement on Patients and Methods**

**A. Retrieval of patient data from the TCGA database**

Patient demographics (age, gender, sex), the American Joint Committee on Cancer (AJCC, 7^th^ edition) stage at original melanoma diagnosis, and the AJCC stage at specimen procurement corresponding to the tumor specimen submitted to TCGA for analysis (“tumor_stage” and “submitted_tumor_site”) were retrieved from the TCGA File Transfer Protocol (FTP) server ([https://tcga-data.nci.nih.gov/tcgafiles/ftp_auth/distro_ftpusers/ anonymous/tumor/skcm](https://tcga-data.nci.nih.gov/tcgafiles/ftp_auth/distro_ftpusers/%20anonymous/tumor/skcm) on September 21, 2015). Latest clinical follow-up (“days_to_last_follow_up” and “days_to_death”) from the time of patients’ original diagnosis of cutaneous melanoma (SKCM) was obtained from the National Cancer Institute (NCI) Genomic Data Commons Data Portal (https://gdc-portal.nci.nih.gov/, Data Release 1.0, June 6, 2016). Patients (“submitter_id”) corresponding to the procured specimens used in this study were censored at the time of the last follow-up (“vital_status”).

**B. Classification of TCGA tumor samples according to the previously described gene expression profiles**

TCGA SKCM gene expression within-sample upper quartile-normalized and per-gene median-centered RNA-Seq by Expectation-Maximization (RSEM) data [1] were downloaded from the TCGA data portal. We then used the previously published top 1,500 genes and subtypes [2] to calculate the silhouette width for each sample [3]. Samples with positive silhouette width were considered to be core samples and were used to define a gene expression predictor of the three RNA expression-based clusters using Classification to the Nearest Centroids (CLaNC) [4]. A signature of 1,260 genes was developed with a 7.2% cross validation error rate and a 5% training error rate. This signature was then applied to the entire SKCM TCGA tumor sample cohort to classify samples into “immune-high”, “microphthalmia-associated transcription factor (MITF)-low”, and “keratin-high” subtypes.

**C. Multi-step filters for somatic mutation calling**

The following multi-step filters for somatic mutation calling were applied to identify non-synonymous mutations in potential melanoma driver genes. First, mutations must have passed the filters [annotated as “PASS” in column “FILTER”; i.e. excluding mutations annotated to be filtered out by both MuTect v1 and 8-oxoguanine (OxoG) Artifact Filter v3 in the variant call format (vcf) files] provided in the vcf files by the Broad Institute and have an allele frequency of no more than 10^-3^, if reported in ExAC (v0.3). Second, their corresponding genes must have been included in the CANCER CENSUS gene list (http://cancer.sanger.ac.uk/census/) by the COSMIC database (v77). To be considered biologically significant mutations, they should be reported in COSMIC with either HIGH (e.g. frameshift, splice site, start loss, stop gain/loss) or MODERATE (e.g. missense) impact by snpEFF (http://snpeff.sourceforge.net/SnpEff_manual.html). Third, single nucleotide variants were re-annotated and merged as dinucleotide mutations, if they were located within the same codon of a sample and their mutated allele frequencies (MAF) were <0.05 apart.

**Supplement on Results**

### Characterization of somatic mutations in the 9 somatically mutated genes that were promising for prognostic significance in stage III/IV SKCM TCGA dataset

**Fig S4.** shows all somatic mutations according to type, survival status, and relation to different domains of each protein corresponding to each of the 9 genes that were found to be prognostically significant in the OS analysis when time from original diagnosis of melanoma was considered in the TCGA dataset. 18 out of 25 observed mutations in the *RAC1* gene were located in codon 29 and 1 mutation in the recently described alternative *RAC1* mutational hotspot, A159 [5]; 11 of these 18 P29 codon mutations were present in specimens from patients who were deceased at the time of analysis.

18 of the 21 observed *MAP2K1* mutations were structural interaction variants. These were calculated from single protein databank entries, by selecting amino acids that are an atom within 3 Ångström (Å) of each other and are at least 20 amino acids away in the amino acid sequence. The assumption is that their close proximity suggests that they must be “interacting”, and thus important for protein structure. 13 of these 18 mutations were found in codon 124 (n=10) and 203 (n=3). However, mutations in codons 56 (n=1) and 57 (n=2), which are more commonly found in lung adenocarcinomas [6], were less frequent in SKCM (n=3, total). With a few exceptions, somatic mutations that were seen in each of the remaining 7 genes (*AFF4, SPEN, CUX1, TSC2, CNTRL, KDM5A, AKAP9*) were unique and were similarly evenly distributed across the entire protein.

**Supplemental Figures**

**Fig S1. Filtering strategy for high-quality somatic mutations according to mutated allele count (MAC), mutated allele frequency (MAF), and coverage depth (DP).** A contour plot superimposed on a scatterplot of mutated allele count (MAC) against total read depth of 5,698 mutations before applying filtering over MAC and MAF. The somatic mutations that were included in the final analysis (n=5,351), shown in filled gray circles (•), are located in the upper left quadrant that is formed by the horizontal black line that separates mutations into those with MAC < 5 and ≥ 5 as well as the oblique red line that separates mutations with MAF (MAC/DP) < 0.05 and ≥ 0.05. Mutations that were filtered out (n=347) are shown in open gray circles (°). Several other oblique and horizontal lines, which represent different levels of MAF, DP, and MAC, are also shown. Contour lines were drawn for mutation concentrations from 10% to 90% in 10% intervals.

**Fig S2. Mutated allele count (MAC), mutated allele frequency (MAF), and coverage depth (DP) of *BRAF*, *RAS*, and *NF1* mutations in the TCGA SKCM dataset.** See S1 Fig legend for details.

**Fig S3. Mean allele frequency (MAF) of somatic mutations in the 22 prognostically significant genes in relation to *BRAF*V600 mutations.** Mutated genes with FDR<0.2 are shown in red **A (upper)**. Metastatic melanoma samples only. Results are shown as boxplots with median and 25/75 percentiles. Asterisks show significant differences at different adjusted p-value (FDR) levels (Wilcoxon test; +, p=0.05-0.1; *, p=0.01-0.05; **, p=0.001-0.01; ***, p=0.0001-0.001). **B (lower)**. Comparison of MAF for *BRAFV600* mutations and the somatically mutated 22 genes in primary (PrM, n=113) versus metastatic melanoma samples (Met, n=356). Results are shown as counts per 100 patients (pt).


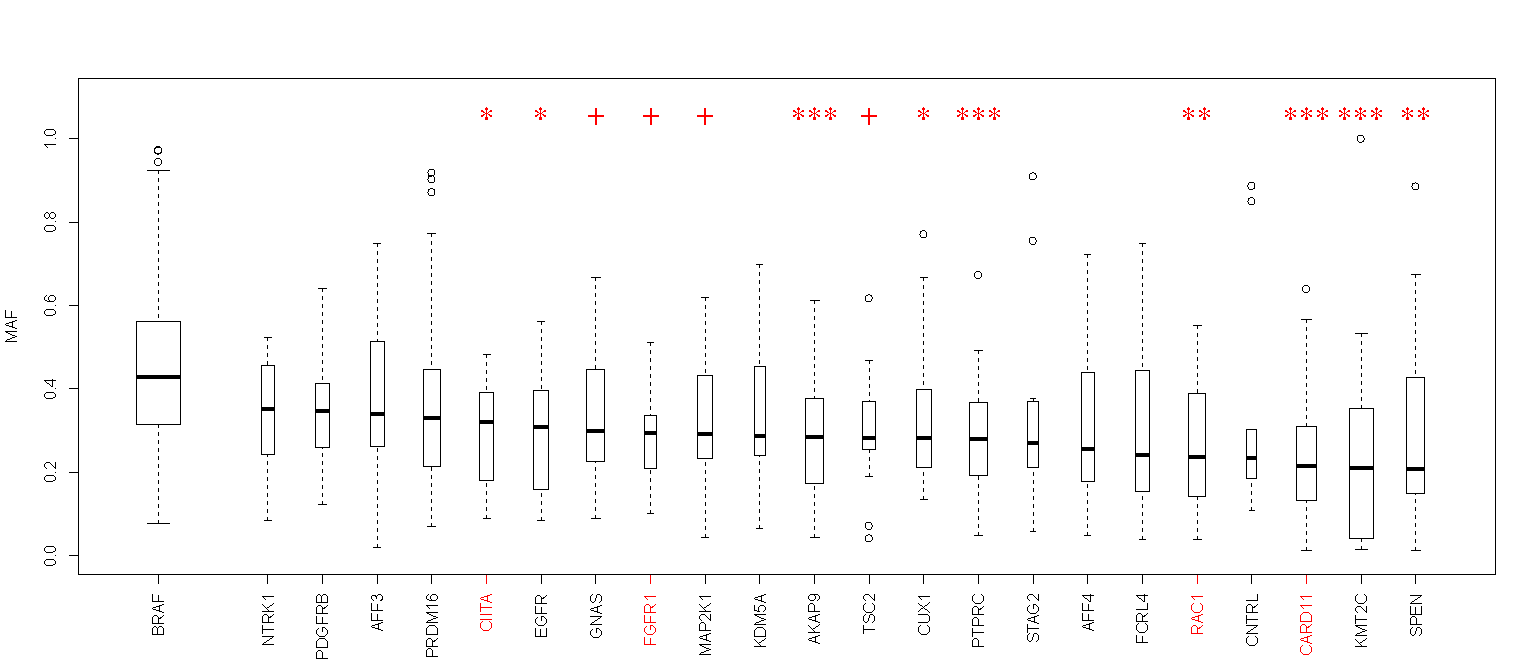


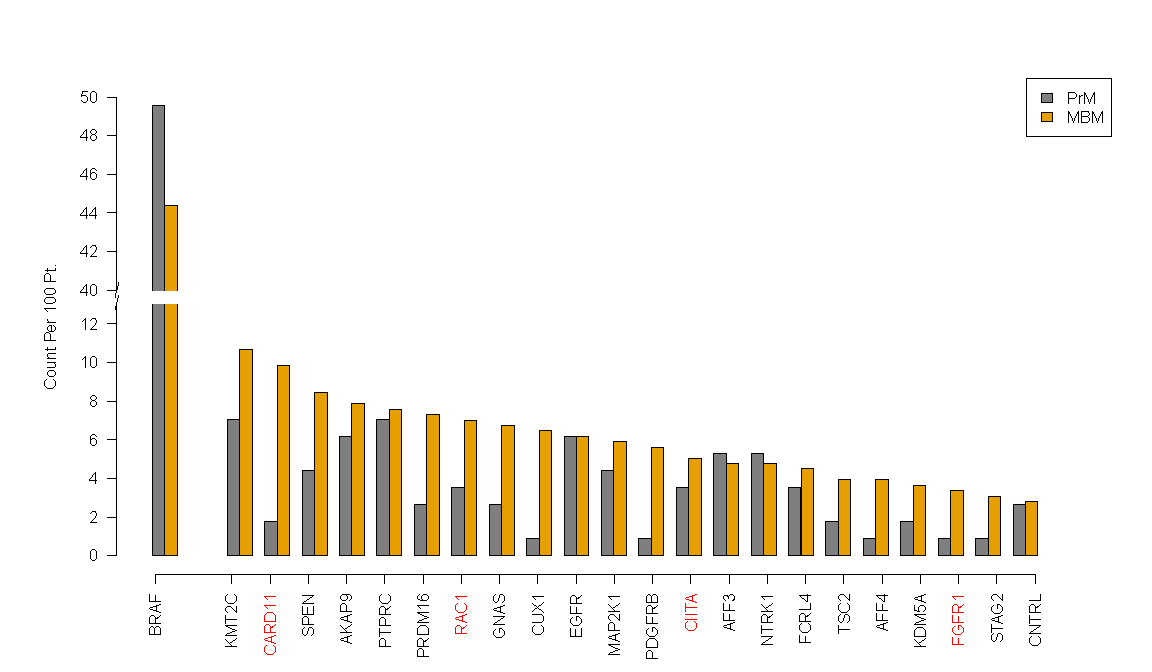


**Fig S4. Distribution of non-synonymous somatic mutations in relation to different domains of each protein encoded by each of the 9 gene as seen in the TCGA SKCM cohort.** Somatic mutations are further highlighted according to their type (missense, red; nonsense, black; structural interaction variant, blue; splice, orange; stop-loss, grey; protein-protein contact, yellow) and survival status (alive, circle; deceased, square).

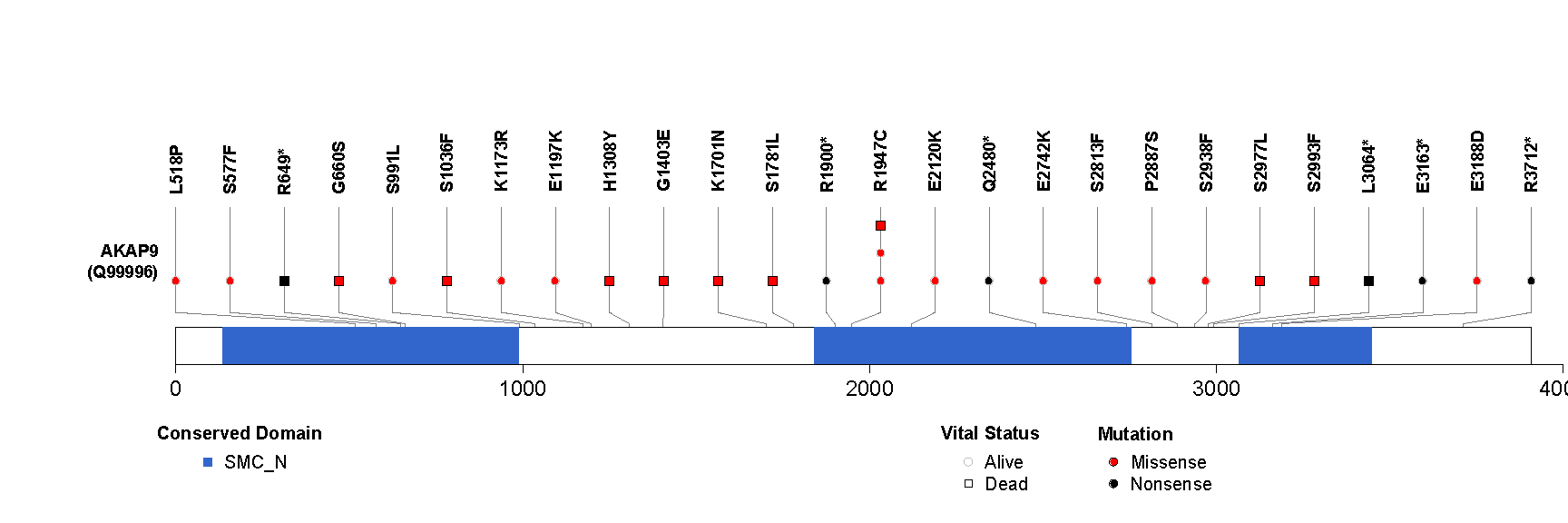


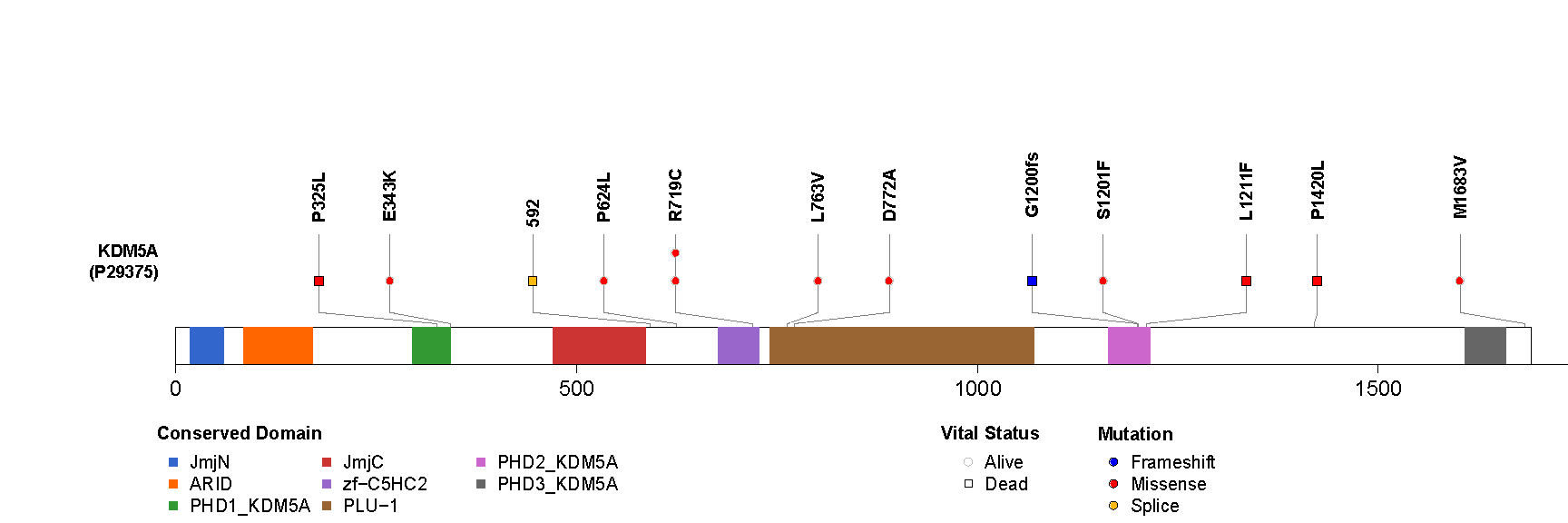


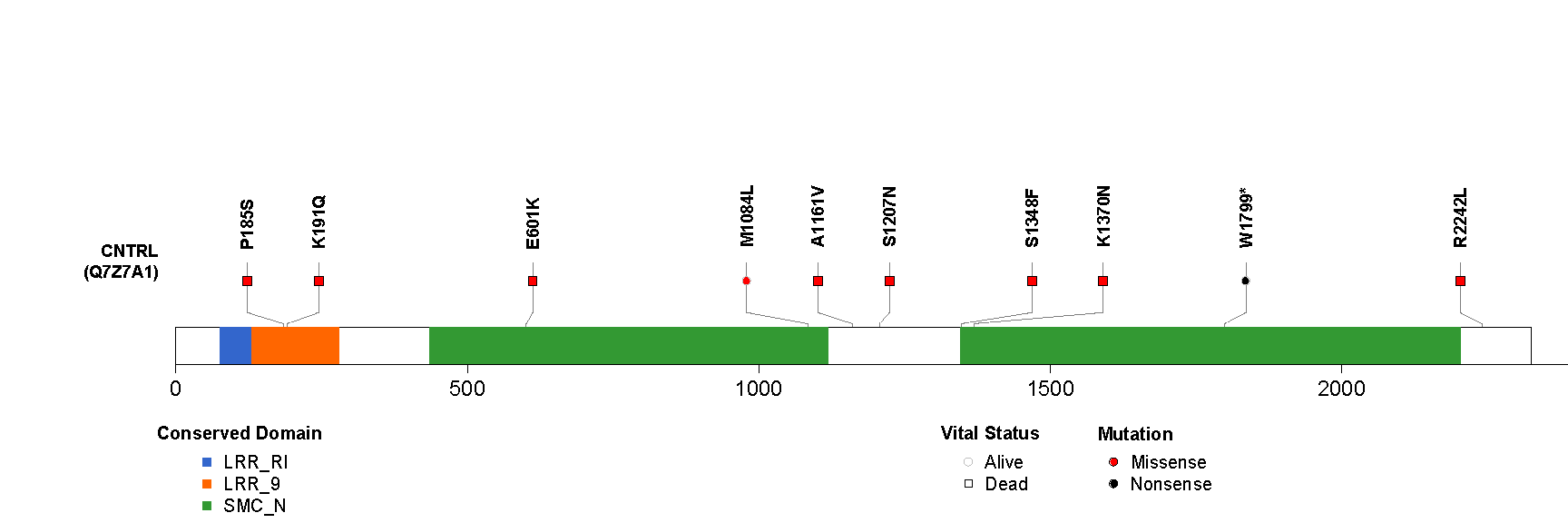

**Supplemental Tables**

**Table S1. Genes mutated in no less than 11 (3%) of samples**. All genes were significantly mutated compared to the background mutation rate (binomial FDR value <10^-8^). Number of specimens with mutated genes of interest are shown separately in deceased versus living subjects. Fold in the normalized frequency of samples mutated in deceased versus living patients is shown based on the formula:

($x=\frac{(No pts with a given mutated gene in deceased)/living pts}{\frac{No pts with a given mutated gene in living pts}{deceased pts}}$ ).

For mutated genes with no less than 50% change in deceased versus living subjects, point estimates with confidence intervals (p-value and FDR) for hazard ratios are reported when OS time from original diagnosis was used to perform Cox analysis (n=53, **panel A**). **Panel B** shows genes with less than 50% change in deceased versus living subjects when OS from time from original diagnosis was used to perform Cox analysis. **Panel C** shows 5 significantly mutated genes that were additionally seen in the cohort of 363 patients when OS time from specimen collection was used to perform Cox analysis. All other genes from Panel A and Panel B were also seen in the 363-patient cohort. **Panel D** shows mutated genes with no less than 50% change in deceased versus living subjects, point estimates with confidence intervals (p-value and FDR) for hazard ratios are reported when OS time from time from specimen biopsy was used to perform Cox analysis (n=57). Highlighted in red and blue are mutated genes with FDR <0.2, and 0.2-0.4, respectively. Highlighted in green are mutated genes that have been previously published as clinically important but had low prognostic significance based on our analysis. Highlighted in pink are mutated genes that had potential prognostic significance in the OS-original diagnosis analysis but ultimately did not have prognostic significance for the OS-time from specimen collection analysis. *Abbreviations*: exp(coef), hazard ratio; cox.pvalue, P value in the Cox hazard ratio analysis; cox.fdr, false discovery rate in the Cox hazard ratio analysis.

**Panel A**

**Gene No samples with Mutated Fold change exp(coef) Cox Cox.fdr**

**Gene of interest (dead/alive) (CI, lower, upper) p-value**

|  |  | **All** | **Dead** | **Alive** |  | |  | |  | |  | |
| --- | --- | --- | --- | --- | --- | --- | --- | --- | --- | --- | --- | --- |
| 1 | *CIITA* | 18 | 2 | 16 | 0.12 | 0.18, (0.04, 0.69) | | 0.01 | | **0.17** | |  |
| 2 | *FGFR1* | 11 | 9 | 2 | 4.16 | 3.01, (1.53, 5.94) | | <0.01 | | **0.08** | |  |
| 3 | *DCTN1* | 12 | 3 | 9 | 0.31 | 0.50, (0.16, 1.58) | | 0.24 | | 0.44 | |  |
| 4 | *STAG2* | 11 | 3 | 8 | 0.35 | 0.50, (0.16, 1.55) | | 0.23 | | 0.44 | |  |
| 5 | *BCOR* | 11 | 3 | 8 | 0.35 | 1.34, (0.43, 4.19) | | 0.62 | | 0.74 | |  |
| 6 | *CUX1* | 18 | 5 | 13 | 0.36 | 0.45, (0.19, 1.11) | | 0.08 | | **0.36** | |  |
| 7 | *TSC2* | 12 | 9 | 3 | 2.77 | 1.95, (0.99, 3.82) | | 0.05 | | **0.27** | |  |
| 8 | *NCOR1* | 19 | 14 | 5 | 2.59 | 1.51, (0.88, 2.62) | | 0.14 | | 0.44 | |  |
| 9 | *BRIP1* | 11 | 8 | 3 | 2.46 | 1.37, (0.67, 2.79) | | 0.39 | | 0.55 | |  |
| 10 | *EP300* | 13 | 4 | 9 | 0.41 | 0.49, (0.18, 1.32) | | 0.16 | | 0.44 | |  |
| 11 | *CBLB* | 13 | 4 | 9 | 0.41 | 0.71, (0.26, 1.92) | | 0.50 | | 0.68 | |  |
| 12 | *PRDM16* | 22 | 7 | 15 | 0.43 | 0.62, (0.29, 1.32) | | 0.21 | | 0.44 | |  |
| 13 | *AFF4* | 14 | 10 | 4 | 2.31 | 1.97, (1.04, 3.75) | | 0.04 | | **0.27** | |  |
| 14 | *NONO* | 12 | 4 | 8 | 0.46 | 0.54, (0.20, 1.46) | | 0.23 | | 0.44 | |  |
| 15 | *EGFR* | 20 | 14 | 6 | 2.16 | 1.60, (0.93, 2.77) | | 0.09 | | **0.37** | |  |
| 16 | *CARD11* | 29 | 10 | 19 | 0.49 | 0.41, (0.21, 0.77) | | 0.01 | | **0.11** | |  |
| 17 | *FGFR2* | 26 | 9 | 17 | 0.49 | 0.66, (0.34, 1.30) | | 0.23 | | 0.44 | |  |
| 18 | *TET1* | 22 | 15 | 7 | 1.98 | 1.09, (0.63, 1.90) | | 0.75 | | 0.83 | |  |
| 19 | *BCL9* | 14 | 5 | 9 | 0.51 | 0.65, (0.27, 1.58) | | 0.34 | | 0.51 | |  |
| 20 | *BRD4* | 14 | 5 | 9 | 0.51 | 0.89, (0.37, 2.17) | | 0.80 | | 0.85 | |  |
| 21 | *PTPRC* | 25 | 9 | 16 | 0.52 | 0.57, (0.29, 1.11) | | 0.10 | | **0.37** | |  |
| 22 | *GNAS* | 22 | 8 | 14 | 0.53 | 0.63, (0.31, 1.28) | | 0.20 | | 0.44 | |  |
| 23 | *IKZF1* | 11 | 4 | 7 | 0.53 | 0.67, (0.25, 1.80) | | 0.42 | | 0.59 | |  |
| 24 | *PBX1* | 11 | 4 | 7 | 0.53 | 0.83, (0.31, 2.23) | | 0.71 | | 0.81 | |  |
| 25 | *SYK* | 12 | 8 | 4 | 1.85 | 1.55, (0.76, 3.15) | | 0.23 | | 0.44 | |  |
| 26 | *TET2* | 12 | 8 | 4 | 1.85 | 1.41, (0.69, 2.88) | | 0.34 | | 0.51 | |  |
| 27 | *AKAP9* | 27 | 10 | 17 | 0.54 | 0.67, (0.34, 1.32) | | 0.25 | | 0.44 | |  |
| 28 | *FCRL4* | 16 | 6 | 10 | 0.55 | 0.45, (0.20, 1.02) | | 0.06 | | **0.27** | |  |
| 29 | *PRDM1* | 16 | 6 | 10 | 0.55 | 0.61, (0.27, 1.39) | | 0.24 | | 0.44 | |  |
| 30 | *RET* | 16 | 6 | 10 | 0.55 | 0.64, (0.26, 1.57) | | 0.33 | | 0.51 | |  |
| 31 | *SLC34A2* | 16 | 6 | 10 | 0.55 | 0.87, (0.39, 1.98) | | 0.75 | | 0.83 | |  |
| 32 | ***CDKN2A*** | 45 | 17 | 28 | 0.56 | 0.72, (0.44, 1.20) | | 0.21 | | 0.44 | |  |
| 33 | *SRGAP3* | 29 | 11 | 18 | 0.56 | 0.86, (0.47, 1.58) | | 0.63 | | 0.74 | |  |
| 34 | *MAP2K1* | 21 | 8 | 13 | 0.57 | 0.46, (0.22, 0.93) | | 0.03 | | **0.27** | |  |
| 35 | *KDM5A* | 13 | 5 | 8 | 0.58 | 0.54, (0.20, 1.46) | | 0.23 | | 0.44 | |  |
| 36 | *PTPRB* | 57 | 37 | 20 | 1.71 | 1.12, (0.77, 1.63) | | 0.55 | | 0.71 | |  |
| 37 | *KDR* | 41 | 16 | 25 | 0.59 | 0.75, (0.45, 1.26) | | 0.28 | | 0.47 | |  |
| 38 | *KMT2C* | 31 | 20 | 11 | 1.68 | 1.31, (0.82, 2.10) | | 0.26 | | 0.44 | |  |
| 39 | *POLE* | 14 | 9 | 5 | 1.66 | 0.99, (0.50, 1.94) | | 0.97 | | 0.98 | |  |
| 40 | *RAC1* | 25 | 16 | 9 | 1.64 | 2.10, (1.25, 3.55) | | 0.01 | | **0.11** | |  |
| 41 | *ATR* | 15 | 6 | 9 | 0.62 | 0.79, (0.35, 1.79) | | 0.58 | | 0.73 | |  |
| 42 | *PTPN13* | 11 | 7 | 4 | 1.62 | 1.81, (0.84, 3.87) | | 0.13 | | 0.44 | |  |
| 43 | *ZNF331* | 19 | 12 | 7 | 1.58 | 1.56, (0.86, 2.81) | | 0.14 | | 0.44 | |  |
| 44 | ***ARID2*** | 38 | 24 | 14 | 1.58 | 1.04, (0.67, 1.62) | | 0.85 | | 0.89 | |  |
| 45 | *PDGFRB* | 19 | 12 | 7 | 1.58 | 1.01, (0.55, 1.83) | | 0.98 | | 0.98 | |  |
| 46 | *SPEN* | 27 | 11 | 16 | 0.64 | 0.55, (0.30, 1.01) | | 0.05 | | **0.27** | |  |
| 47 | *AFF3* | 16 | 10 | 6 | 1.54 | 1.95, (1.02, 3.74) | | 0.04 | | **0.27** | |  |
| 48 | *NTRK1* | 16 | 10 | 6 | 1.54 | 1.93, (1.02, 3.68) | | 0.04 | | **0.27** | |  |
| 49 | *CACNA1D* | 24 | 15 | 9 | 1.54 | 1.33, (0.78, 2.26) | | 0.30 | | 0.48 | |  |
| 50 | *UBR5* | 16 | 10 | 6 | 1.54 | 1.10, (0.58, 2.10) | | 0.77 | | 0.83 | |  |
| 51 | *RAF1* | 12 | 5 | 7 | 0.66 | 0.52, (0.21, 1.28) | | 0.16 | | 0.44 | |  |
| 52 | ***FBXW7*** | 12 | 5 | 7 | 0.66 | 0.76, (0.31, 1.85) | | 0.54 | | 0.71 | |  |
| 53 | *CIC* | 12 | 5 | 7 | 0.66 | 1.28, (0.52, 3.13) | | 0.59 | | 0.73 | |  |

**Panel B**

**No samples with Fold No samples with Fold**

**Gene Mutated Gene Change Gene Mutated Gene Change**

**of Interest (dead/alive) of Interest (dead/alive)**

**All Dead Alive All Dead Alive**

| **54** | *MTOR* | 19 | 8 | 11 | 0.67 | | 92 | | *FLT3* | | 31 | 15 | | 16 | | | 0.87 | | |  |  |
| --- | --- | --- | --- | --- | --- | --- | --- | --- | --- | --- | --- | --- | --- | --- | --- | --- | --- | --- | --- | --- | --- |
| **55** | *SETD2* | 13 | 8 | 5 | 1.48 | | 93 | | *FAT4* | | 101 | 56 | | 45 | | | 1.15 | | |  |  |
| **56** | *CBL* | 13 | 8 | 5 | 1.48 | | 94 | | *MECOM* | | 65 | 36 | | 29 | | | 1.15 | | |  |  |
| **57** | *NUP98* | 18 | 11 | 7 | 1.45 | | 95 | | *LIFR* | | 35 | 17 | | 18 | | | 0.87 | | |  |  |
| **58** | *SMARCA4* | 21 | 9 | 12 | 0.69 | | 96 | | *PDE4DIP* | | 41 | 20 | | 21 | | | 0.88 | | |  |  |
| **59** | *FLT4* | 21 | 9 | 12 | 0.69 | | 97 | | *RNF213* | | 22 | 12 | | 10 | | | 1.11 | | |  |  |
| **60** | *COL1A1* | 33 | 20 | 13 | 1.42 | | 98 | | *RUNX1T1* | | 33 | 18 | | 15 | | | 1.11 | | |  |  |
| **61** | *ELN* | 16 | 7 | 9 | 0.72 | | 99 | | *PBRM1* | | 11 | 6 | | 5 | | | 1.11 | | |  |  |
| **62** | *MET* | 20 | 12 | 8 | 1.39 | | 100 | | *PTPRK* | | 37 | 20 | | 17 | | | 1.09 | | |  |  |
| **63** | *NUTM1* | 25 | 11 | 14 | 0.73 | | 101 | | *PPARG* | | 12 | 6 | | 6 | | | 0.92 | | |  |  |
| **64** | *MYH11* | 27 | 12 | 15 | 0.74 | | 102 | | ***ARID1B*** | | 16 | 8 | | 8 | | | 0.92 | | |  |  |
| **65** | ***TP53*** | 56 | 25 | 31 | 0.75 | | 103 | | *NUP214* | | 12 | 6 | | 6 | | | 0.92 | | |  |  |
| **66** | *PDGFRA* | 22 | 13 | 9 | 1.34 | | 104 | | *BCL11B* | | 18 | 9 | | 9 | | | 0.92 | | |  |  |
| **67** | *DICER1* | 20 | 9 | 11 | 0.76 | | 105 | | *CTNNB1* | | 18 | 9 | | 9 | | | 0.92 | | |  |  |
| **68** | *CHD4* | 17 | 10 | 7 | 1.32 | | 106 | | *AMER1* | | 20 | 10 | | 10 | | | 0.92 | | |  |  |
| **69** | *IL7R* | 31 | 14 | 17 | 0.76 | | 107 | | *CASC5* | | 12 | 6 | | 6 | | | 0.92 | | |  |  |
| **70** | ***PPP6C*** | 22 | 10 | 12 | 0.77 | | 108 | | *SETBP1* | | 24 | 12 | | 12 | | | 0.92 | | |  |  |
| **71** | *KMT2D* | 33 | 15 | 18 | 0.77 | | 109 | | ***PTEN*** | | 24 | 12 | | 12 | | | 0.92 | | |  |  |
| **72** | *NCOA2* | 11 | 5 | 6 | 0.77 | | 110 | | *ARID1A* | | 14 | 7 | | 7 | | | 0.92 | | |  |  |
| **73** | *MLLT4* | 12 | 7 | 5 | 1.29 | | 111 | | *SF3B1* | | 16 | 8 | | 8 | | | 0.92 | | |  |  |
| **74** | *KIAA1549* | 24 | 14 | 10 | 1.29 | | 112 | | *ATRX* | | 14 | 7 | | 7 | | | 0.92 | | |  |  |
| **75** | ***NF1*** | 36 | 21 | 15 | 1.29 | | 113 | | *NCOR2* | | 16 | 8 | | 8 | | | 0.92 | | |  |  |
| **76** | *LCK* | 13 | 6 | 7 | 0.79 | | 114 | | *RANBP2* | | 22 | 11 | | 11 | | | 0.92 | | |  |  |
| **77** | *NSD1* | 13 | 6 | 7 | 0.79 | | 115 | | *CAMTA1* | | 22 | 11 | | 11 | | | 0.92 | | |  |  |
| **78** | *KAT6A* | 13 | 6 | 7 | 0.79 | | 116 | | ***KIT*** | | 13 | 7 | | 6 | | | 1.08 | | |  |  |
| **79** | ***NRAS*** | 106 | 61 | 45 | 1.25 | | 117 | | *NRG1* | | 13 | 7 | | 6 | | | 1.08 | | |  |  |
| **80** | *ATM* | 14 | 8 | 6 | 1.23 | | 118 | | *APC* | | 26 | 14 | | 12 | | | 1.08 | | |  |  |
| **81** | *ROS1* | 56 | 32 | 24 | 1.23 | | 119 | | *TRRAP* | | 41 | 22 | | 19 | | | 1.07 | | |  |  |
| **82** | *ZFHX3* | 35 | 20 | 15 | 1.23 | | 120 | | ***BRAF*** | | 179 | 90 | | 89 | | | 0.93 | | |  |  |
| **83** | ***NOTCH2*** | 14 | 8 | 6 | 1.23 | | 121 | | *COL2A1* | | 28 | 15 | | 13 | | | 1.07 | | |  |  |
| **84** | *CREBBP* | 19 | 9 | 10 | 0.83 | | 122 | | *ALK* | | 28 | 15 | | 13 | | | 1.07 | | |  |  |
| **85** | *GRIN2A* | 82 | 39 | 43 | 0.84 | | 123 | | *ACSL6* | | 15 | 8 | | 7 | | | 1.06 | | |  |  |
| **86** | *NTRK3* | 21 | 10 | 11 | 0.84 | | 124 | | *IDH1* | | 17 | 9 | | 8 | | | 1.04 | | |  |  |
| **87** | *BRCA2* | 16 | 9 | 7 | 1.19 | | 125 | | *IL21R* | | 17 | 9 | | 8 | | | 1.04 | | |  |  |
| **88** | *ERBB4* | 50 | 24 | 26 | 0.85 | | 126 | | *USP6* | | 21 | 11 | | 10 | | | 1.02 | | |  |  |
| **89** | *BCL11A* | 25 | 12 | 13 | 0.85 | | 127 | | *ITK* | | 23 | 12 | | 11 | | | 1.01 | | |  |  |
| **90** | *KMT2A* | 34 | 19 | 15 | 1.17 | | 128 | | *FAT1* | | 25 | 13 | | | 12 | | | | 1.00 | |  |
| 91 | *RANBP17* | 18 | 10 | 8 | 1.16 |  | |  | |  | | |  | | |  | |  | | | |

**Panel C**

**No samples with Fold**

**Gene Mutated Gene Change**

**Of Interest (dead/alive)**

|  |  | **All** | | **Dead** | **Alive** | |  |  |  |  |
| --- | --- | --- | --- | --- | --- | --- | --- | --- | --- | --- |
| **129** | *CNTRL* | | | 11 | 9 | | | 2 | | 0.67 |
| **130** | *WRN* | | | 11 | 7 | | | 4 | | 1.48 |
| **131** | *MAP3K13* | | | 11 | 3 | | | 8 | | 1.48 |
| **132** | *ZBTB16* | | | 11 | 6 | | | 5 | |  |
| **133** | *HIP1* | | | 11 | 7 | | | 4 | |  |

**Panel D**

**No samples with Mutated Fold change exp(coef) Cox Cox.fdr**

**Gene of interest (dead/alive) (CI, lower, upper) p-value**

**All Dead Alive**

| 1 | *FGFR1* | 11 | 9 | 2 | 4.16 | 2.70 (1.38, 5.30) | <0.01 | 0.22 |
| --- | --- | --- | --- | --- | --- | --- | --- | --- |
| 2 | *CIITA* | 18 | 2 | 16 | 0.13 | 0.18 (0.04, 0.69) | 0.01 | 0.36 |
| 3 | *CNTRL* | 11 | 9 | 2 | 4.50 | 2.07 (1.06, 4.06) | 0.03 | 0.38 |
| 4 | *GNAS* | 22 | 8 | 14 | 0.53 | 0.48 (0.24, 0.98) | 0.04 | 0.38 |
| 5 | *AKAP9* | 28 | 10 | 18 | 0.54 | 0.50 (0.26, 0.99) | 0.05 | 0.38 |
| 6 | *KMT2C* | 32 | 21 | 11 | 1.68 | 1.58 (0.99, 2.51) | 0.05 | 0.38 |
| 7 | *PRDM16* | 22 | 7 | 15 | 0.43 | 0.48 (0.22, 1.02) | 0.06 | 0.38 |
| 8 | *CARD11* | 29 | 10 | 19 | 0.49 | 0.54 (0.28, 1.03) | 0.06 | 0.38 |
| 9 | *SPEN* | 29 | 12 | 17 | 0.64 | 0.58 (0.32, 1.04) | 0.07 | 0.38 |
| 10 | *KDM5A* | 13 | 5 | 8 | 0.58 | 0.41 (0.15, 1.09) | 0.07 | 0.38 |
| 11 | *PDGFRB* | 19 | 12 | 7 | 1.58 | 1.64 (0.91, 2.95) | 0.10 | 0.38 |
| 12 | *STAG2* | 11 | 3 | 8 | 0.35 | 0.39 (0.12, 1.21) | 0.10 | 0.38 |
| 13 | *SYK* | 12 | 8 | 4 | 1.85 | 1.78 (0.87, 3.62) | 0.11 | 0.38 |
| 14 | *AFF4* | 14 | 10 | 4 | 2.31 | 1.68 (0.89, 3.17) | 0.11 | 0.38 |
| 15 | *FGFR2* | 27 | 10 | 17 | 0.49 | 0.60 (0.32, 1.14) | 0.12 | 0.38 |
| 16 | *DCTN1* | 12 | 3 | 9 | 0.31 | 0.41 (0.13, 1.28) | 0.13 | 0.38 |
| 17 | *NCOR1* | 19 | 14 | 5 | 2.59 | 1.51 (0.88, 2.61) | 0.14 | 0.38 |
| 18 | *EP300* | 13 | 4 | 9 | 0.41 | 0.47 (0.18, 1.27) | 0.14 | 0.38 |
| 19 | ***RAC1*** | 25 | 16 | 9 | 1.64 | 1.47 (0.88, 2.46) | 0.14 | 0.38 |
| 20 | ***MAP2K1*** | 21 | 8 | 13 | 0.57 | 0.59 (0.29, 1.19) | 0.14 | 0.38 |
| 21 | *FCRL4* | 16 | 6 | 10 | 0.55 | 0.55 (0.24, 1.23) | 0.14 | 0.38 |
| 22 | ***EGFR*** | 21 | 15 | 6 | 2.16 | 1.48 (0.87, 2.50) | 0.15 | 0.38 |
| 23 | *WRN* | 11 | 7 | 4 | 1.75 | 1.72 (0.81, 3.66) | 0.16 | 0.39 |
| 24 | *PTPRC* | 26 | 9 | 17 | 0.52 | 0.63 (0.32, 1.22) | 0.17 | 0.39 |
| 25 | *PRDM1* | 16 | 6 | 10 | 0.55 | 0.57 (0.25, 1.29) | 0.18 | 0.39 |
| 26 | *PTPN13* | 13 | 9 | 4 | 1.62 | 1.59 (0.81, 3.12) | 0.18 | 0.39 |
| 27 | ***CDKN2A*** | 45 | 17 | 28 | 0.56 | 0.73 (0.44, 1.20) | 0.22 | 0.45 |
| 28 | *NONO* | 12 | 4 | 8 | 0.46 | 0.54 (0.20, 1.46) | 0.22 | 0.45 |
| 29 | *IKZF1* | 11 | 4 | 7 | 0.53 | 0.55 (0.20, 1.48) | 0.23 | 0.45 |
| 30 | *NTRK1* | 16 | 10 | 6 | 1.54 | 1.46 (0.77, 2.77) | 0.24 | 0.45 |
| 31 | *MAPK3K13* | 11 | 3 | 8 | 0.38 | 0.51 (0.16, 1.59) | 0.25 | 0.45 |
| 32 | *BCL9* | 14 | 5 | 9 | 0.51 | 0.56 (0.21, 1.52) | 0.26 | 0.46 |
| 33 | *PTPRB* | 58 | 37 | 21 | 1.71 | 1.23 (0.85, 1.77) | 0.27 | 0.46 |
| 34 | *CUX1* | 19 | 6 | 13 | 0.36 | 0.64 (0.28, 1.44) | 0.28 | 0.46 |
| 35 | *RAF1* | 12 | 5 | 7 | 0.66 | 0.61 (0.25, 1.50) | 0.28 | 0.46 |
| 36 | *TSC2* | 12 | 9 | 3 | 2.77 | 1.44 (0.74, 2.82) | 0.29 | 0.46 |
| 37 | ***ARID2*** | 38 | 24 | 14 | 1.58 | 1.25 (0.81, 1.92) | 0.32 | 0.49 |
| 38 | *TET2* | 13 | 9 | 4 | 1.85 | 1.38 (0.71, 2.70) | 0.35 | 0.52 |
| 39 | *CBLB* | 13 | 4 | 9 | 0.41 | 0.65 (0.24, 1.77) | 0.40 | 0.57 |
| 40 | *RET* | 17 | 7 | 10 | 0.55 | 0.71 (0.31, 1.59) | 0.40 | 0.57 |
| 41 | *MTOR* | 19 | 8 | 11 | 0.72 | 0.75 (0.37, 1.52) | 0.42 | 0.59 |
| 42 | *SRGAP3* | 29 | 11 | 18 | 0.56 | 0.79 (0.43, 1.46) | 0.46 | 0.61 |
| 43 | *BRIP1* | 11 | 8 | 3 | 2.46 | 1.30 (0.64, 2.66) | 0.46 | 0.61 |
| 44 | *CACNA1D* | 25 | 16 | 9 | 1.54 | 1.20 (0.72, 2.00) | 0.49 | 0.63 |
| 45 | *SLC34A2* | 16 | 6 | 10 | 0.55 | 0.76 (0.34, 1.71) | 0.51 | 0.64 |
| 46 | *ATR* | 15 | 6 | 9 | 0.62 | 0.80 (0.35, 1.81) | 0.59 | 0.73 |
| 47 | *KDR* | 42 | 17 | 25 | 0.59 | 0.89 (0.54, 1.46) | 0.63 | 0.76 |
| 48 | *TET1* | 22 | 15 | 7 | 1.98 | 1.14 (0.66, 1.97) | 0.64 | 0.76 |
| 49 | *ZNF331* | 19 | 12 | 7 | 1.58 | 1.14 (0.63, 2.04) | 0.67 | 0.76 |
| 50 | *BRD4* | 14 | 5 | 9 | 0.51 | 0.83 (0.34, 2.02) | 0.68 | 0.76 |
| 51 | *FBXW7* | 12 | 5 | 7 | 0.66 | 0.83 (0.34, 2.02) | 0.68 | 0.76 |
| 52 | *PBX1* | 11 | 4 | 7 | 0.53 | 0.86 (0.32, 2.31) | 0.76 | 0.83 |
| 53 | *UBR5* | 16 | 10 | 6 | 1.54 | 1.03 (0.54, 1.95) | 0.93 | 0.99 |
| 54 | *CIC* | 12 | 5 | 7 | 0.66 | 1.03 (0.42, 2.53) | 0.94 | 0.99 |
| 55 | *POLE* | 14 | 9 | 5 | 1.66 | 0.99 (0.50, 1.93) | 0.97 | 1.00 |
| 56 | *HIP1* | 11 | 7 | 4 | 1.75 | 1.00 (0.47, 2.14) | 1.00 | 1.00 |
| 57 | *BCOR* | 11 | 3 | 8 | 0.35 | 1.00 (0.32, 3.13) | 1.00 | 1.00 |

**Table S2. Patient demographics and characteristics of the UNC-CH UNCseq™ AJCC stage III/IV cohort.**

⎯⎯⎯⎯⎯⎯⎯⎯⎯⎯⎯⎯⎯⎯⎯⎯⎯⎯⎯⎯⎯⎯⎯⎯⎯⎯⎯⎯⎯⎯⎯⎯⎯⎯⎯⎯

**Characteristic** **Total (n=33)**

⎯⎯⎯⎯⎯⎯⎯⎯⎯⎯⎯⎯⎯⎯⎯⎯⎯⎯⎯⎯⎯⎯⎯⎯⎯⎯⎯⎯⎯⎯⎯⎯⎯⎯⎯⎯

**Sex, n (%)**

Male 20

Female 13

**Age (years), median (range)** 58 (34-82)

**AJCC stage at specimen collection**

III 13

IV 20

**Systemic treatments after specimen acquisition**

0 8

1 15

2 5

3 3

>3 2

**Molecular classifications**

*BRAF* mutations 14

*BRAF V600/601* 11

*nonBRAF V600/601* 3

*NRAS* codon 12/61 9

*NF1* mutations 6

Stop codon 5

Other 1

**Table S3. Patient characteristics of the UNC-CH MM Cohort whose MM tissues was used for Construction of the MM Tissue Microarray (n=87)**.

**Characteristics** **Total (n=87)**

**Organ Type**

Lymph node or Soft Tissue 80

Lung 6

Liver 1

**Anatomic Site**

Scalp-Neck 29

Trunk 23

Extremeties 27

Unknown Primary 6

Vulva 2

**Age at Original Diagnosis** (years), mean, median, (range) 55.6, 57, (22, 89)

**Sex**

Male 59

Female 28

**Race**

White 82

African American 2

Asian American 1

Hispanic-Latino 2

**References on Data Supplement**

1. Li, B., Dewey, C.N. (2011). RSEM: accurate transcript quantification from RNA-Seq data with or without a reference genome. BMC. Bioinformatics. 12, 323. doi: 10.1186/1471-2105-12-323.

2. Cancer Genome Atlas Network (2015). Genomic Classification of Cutaneous Melanoma. Cell. 161, 1681-1696.

3. Lovmar, L., Ahlford, A., Jonsson, M., Syvanen, A.C. (2005). Silhouette scores for assessment of SNP genotype clusters. BMC. Genomics. 6, 35.

4. Dabney, A.R. (2005). Classification of microarrays to nearest centroids. Bioinformatics. 21, 4148-4154.

5. Chang, M.T., Asthana, S., Gao, S.P., Lee, B.H., Chapman, J.S., et al. (2016). Identifying recurrent mutations in cancer reveals widespread lineage diversity and mutational specificity. Nat. Biotechnol. 34, 155-163.

6. Arcila, M.E., Drilon, A., Sylvester, B.E., Lovly, C.M., Borsu, L., et al. (2015). MAP2K1 (MEK1) Mutations Define a Distinct Subset of Lung Adenocarcinoma Associated with Smoking. Clin. Cancer. Res. 21, 1935-1943.
